# Supplementary material for: Protocol for a human in vivo model of acute cigarette smoke inhalation challenge in smokers with COPD: monitoring the nasal and systemic immune response using a network biology approach
Source: BMJ Open. 2015 Jan 28;5(1):e005750. doi: 10.1136/bmjopen-2014-005750 (PMC4316420; doi:10.1136/bmjopen-2014-005750)
Supplement: Author's manuscript [file bmjopen-2014-005750.draft_revisions.pdf]

BMJ Open

Protocol for a human in vivo model of acute cigarette smoke inhalation challenge in smokers with COPD: monitoring the nasal and systemic immune response using a network biology approach

Journal:	*BMJ Open*
Manuscript ID:	bmjopen-2014-005750
Article Type:	Protocol
Date Submitted by the Author:	27-May-2014
Complete List of Authors:	Ross, Clare; Imperial College Healthcare NHS Trust, Chest and Allergy<br>Galloway-Phillipps, Neil; Imperial College London, Cardiothoracic Pharmocology<br>Armstrong, Paul; Queen Mary's School of Medicine and Dentistry, William Harvey Research Institute<br>Mitchell, Jane; Imperial College London, National Heart and Lung Institute<br>Warner, Timothy; Queen Mary's School of Medicine and Dentistry, William Harvey Research Institute<br>Brearley, Christopher; Sunovion Pharmaceuticals Europe Ltd,<br>Ito, Mari; Dainippon Sumitomo Pharma Co Ltd, ; Tokyo Medical University, Molecular Regulation for intractable Diseases<br>Elkin, Sarah; Imperial College Healthcare NHS Trust, St Mary's Hospital, Respiratory Infection and Medicine, Imperial Clinical Respiratory Research Unit<br>Kon, Onn Min; Imperial College Healthcare NHS Trust, St Mary's Hospital, Respiratory Infection and Medicine, Imperial Clinical Respiratory Research Unit<br>Hansel, Trevor; Imperial College London, Imperial Clinical Respiratory Research Unit (ICRRU) and Biomedical Research Centre (BMRC)<br>Paul-Clark, Mark; Imperial College London, Cardiothoracic Pharmocology; Imperial College HNS Trust, Imperial Clinical Respiratory Research Unit
**Primary Subject Heading**:	Respiratory medicine
Secondary Subject Heading:	Smoking and tobacco, Research methods, Genetics and genomics
Keywords:	Adult thoracic medicine < THORACIC MEDICINE, Chronic airways disease < THORACIC MEDICINE, Emphysema < THORACIC MEDICINE, Immunology < THORACIC MEDICINE

SCHOLARONE™
Manuscripts

Protocol for a human *in vivo* model of acute cigarette smoke inhalation challenge in smokers with COPD: monitoring the nasal and systemic immune response using a network biology approach

Authors:

Clare L. Ross[1] (MRCP) clare.ross@imperial.nhs.uk

Neil Galloway-Phillipps[2] (BSc) n.galloway-phillipps@imperial.ac.uk

Paul C. Armstrong[3] (PhD) p.c.armstrong@qmul.ac.uk

Jane A. Mitchell[2] (PhD) j.a.mitchell@imperial.ac.uk

Timothy D. Warner[3] (PhD) t.d.warner@qmul.ac.uk

Christopher Brearley[4] (MRCP) chris.brearley@sunovion.eu

Mari Ito[5, 6] (PhD) ito-m@osaka-ra-clinic.com

Sarah Elkin[1] (FRCP) sarah.elkin@imperial.nhs.uk

Onn Min Kon[1] (FRCP) onn.kon@imperial.nhs.uk

Trevor T. Hansel[1*] (FRCPath, PhD) t.hansel@imperial.ac.uk

Mark J. Paul-Clark[2*] (PhD) m.paul-clark@imperial.ac.uk

[1]Imperial Clinical Respiratory Research Unit (ICRRU) and Biomedical Research Centre (BMRC), Centre for Respiratory Infection (CRI), St Mary's Hospital, Imperial College, London, UK

[2] National Heart and Lung Institute, Imperial College, London, UK

[3] William Harvey Research Institute, Barts and The London, Queen Mary's School of Medicine and Dentistry, London, UK

[4] Sunovion Pharmaceuticals Europe Ltd. (Sunovion Europe)

[5] Dainippon Sumitomo Pharma Co Ltd, Osaka, Japan

[6] Department of Molecular Regulation for intractable Diseases, Institute of Medical Science, Tokyo Medical University

*Contributed equally to this project

For peer review only

Correspondence to:

Dr Trevor T. Hansel, Imperial Clinical Respiratory Research Unit (ICRRU), St Mary's Hospital, Praed Street, Paddington, London W2 INY, UK

Tel: +44 203 312 5733; email: t.hansel@imperial.ac.uk

Key Words

COPD, human challenge model, cigarette smoke, inflammation, metabolomics, transcriptomics, nicotine, cytokine, network biology, systems biology

Supported by

Dainippon Sumitomo Pharma Co Ltd, Osaka, Japan National Institute of Healthcare Research Biomedical Research Centre (NIHR BMRC)

Imperial Academic Health Science Centre (AHSC)

Imperial Centre for Respiratory Infection (CRI)

For peer review only

ABSTRACT

Introduction: Cigarette smoke contributes to a diverse range of diseases including chronic obstructive pulmonary disease (COPD), cardiovascular disorders and many cancers. There currently is a need for human challenge models, to assess the acute effects of a controlled cigarette smoke stimulus, followed by serial sampling of blood and respiratory tissue for advanced molecular profiling. We employ precision sampling of nasal mucosal lining fluid by absorption to allow for the measurement of soluble mediators in eluate. Serial nasal curettage was used for transcriptomic analysis of mucosal tissue.

Methods and analysis: 3 groups of strictly defined subjects will be studied: 12 smokers with COPD (GOLD stage II) with emphysema, 12 matched smokers with normal lung function and no evidence of emphysema, and 12 matched never smokers with normal spirometry. Subjects in the smoking groups are current smokers, and will be given full support to stop smoking immediately after this study. In giving a controlled cigarette smoke stimulus, all subjects will have abstained from smoking for 12 hours, and will smoke 2 cigarettes with expiration through the nose in a ventilated chamber. Before and after inhalation of cigarette smoke, a series of samples will be taken from the blood, nasal mucosal lining fluid, and nasal tissue by curettage. Analysis of plasma nicotine and metabolites in relation to levels of soluble inflammatory mediators in nasal lining fluid and blood, as well as assessing nasal transcriptomics, *ex vivo* blood platelet aggregation and leukocyte responses to TLR-agonists will be undertaken.

Implications: Development of acute cigarette smoke challenge models has promise for the study of molecular effects of smoking in a range of pathological processes.

Ethics and dissemination: This study was approved by the West London National Research Ethics Committee (12/LO/1101). The study findings will be presented at conferences and will be reported in peer-reviewed journals.

For peer review only

ARTICLE SUMMARY

Article focus:

This study examines the acute effects of cigarette smoke in a carefully selected group of smokers and matched controls, employing a network biology approach utilising non-invasive sampling techniques from the upper respiratory tract in conjunction with serial blood sampling.

Key messages:

Smokers with GOLD Stage II COPD with emphysema on HRCT (aged 45-75) are matched with 'healthy' smokers (with normal lung function and imaging) and non-smoking controls.

A controlled stimulus from smoking 2 cigarettes (with nasal exhalation) will take place in a ventilated chamber following a 12 hour abstinence. There will be subsequent serial nasal and blood sampling over a 5 hour period, during which the subject will refrain from further cigarettes.

Strengths and limitations of the study:

This model involves limited numbers of highly selected individuals, and as such selection bias will inevitably occur. However, in conjunction with serial sampling in controlled conditions, this enables discrimination of relatively small changes in levels of cigarette smoke constituents and the subsequent inflammatory response.

It is possible to study acute effects of cigarette smoke in a variety of smoking-related diseases. In this way it may be possible to gain molecular insight into the pathology of COPD, cardiovascular disease and neoplasia.

BACKGROUND

There are profound causative and contributory effects of chronic cigarette smoking in a range of disease processes, as well as considerable mortality (1;2). In order to understand the changes in the lungs due to chronic smoking, it is relevant to assess the immunological responses to acute smoke exposure. Repetitive acute effects of cigarette smoke in susceptible individuals may lead to cumulative irreversible damage (3). Despite cigarette smoking being the most important risk factor for the development of COPD and a major contributory influence in the development of cardiovascular disease, the use of human cigarette challenge models has been under utilised in the investigation of immune and local influences on the disease (4). This is somewhat surprising, as we still know little about the mechanisms involved in smoke-induced inflammation in man.

Whilst several downstream effects of cigarette smoking are common to all smokers, such as anti-oxidant gene activation and aryl hydrocarbon signaling, it is estimated that only 13-50% of smokers actually develop COPD (5) (6), and within this group,

For peer review only

there is great clinical heterogeneity (2). The immunopathology of COPD is complex and variable, involving the large airways (bronchitis), small airways (bronchiolitis), lung interstitium (emphysema and interstitial lung disease), pulmonary vasculature (pulmonary artery hypertension) and systemic and cardiovascular complications (7). There is also the added complexity attributable to the innate immune response to oxidants and microbes (8) (9). Our group and others have previously demonstrated that cigarette smoke (extract) can activate human immune and respiratory epithelial cells *in vitro* leading to the release of the pro-inflammatory chemokine CXCL8 (10) (11) (12) (13) (14) (15). In addition, following smoking, blood of smokers is 'primed' to activation *ex vivo* by pathogen associated molecular patterns (PAMPs) including LPS (16). Most recently we have performed a pilot transcriptomic study using human monocytes stimulated *in vitro* and shown that smoke activates and inhibits discrete groups of genes involved in oxidant stress and inflammation (17). In addition there have been individual genomic, transcriptomic and metabolomic studies in cell based (18) (19) (20) (21) (22) (23) (24) and animal models (25) of smoking which have further defined the role of cigarette smoke as an inflammatory insult. However, there is now a need for a multi-systems-based approach in man *in vivo* to truly advance our understanding of how cigarette smoking induces inflammation (27).

A protocol for a study of the effects of smoking in patients with chronic obstructive pulmonary disease (COPD) has recently been reported by Tam Loi and colleagues from Utrecht, Netherlands (26). This group proposes the assessment of acute effects of smoking at 5 minutes after smoking 3 cigarettes, at 2 hours, 24 hours and after a 6 week interval. They also propose assessment of cross-sectional inflammatory responses in different patient groups. In this study sampling from patients consists of blood, sputum and exhaled breath condensate (EBC); and they employ endobronchial sampling for biopsy, epithelial lining fluid and epithelial brushings.

Our group is currently conducting an *in vivo* model of acute cigarette smoke inhalation challenge in smokers with COPD and appropriate controls. Our study differs in selection of patients, and having more defined conditions for cigarette smoke exposure. In addition, we have an intensive sampling schedule over the 5 hours following a controlled cigarette smoke stimulus, with a focus on blood and nasal non-invasive sampling, during which we assess levels of nicotine and metabolites in relation to pro-inflammatory effects. Such a study has thus far been difficult since access to human airway tissue and secretion samples in a minimally-invasive serial manner has not previously been possible.

There has been recent progress in finding novel biomarkers for COPD (28;29), and a focus on recognizing new phenotypes of COPD (30). GlaxoSmithKline has completed a 3 year longitudinal study in 2180 COPD subjects entitled ECLIPSE (Evaluation of COPD Longitudinally to Identify Predictive Surrogate Endpoints) (31). In terms of the natural history of COPD it was found that sputum neutrophil counts (32) and exhaled breath condensate (EBC) pH was not useful (33). EBC has limitations of dilution and salivary contamination (34). In contrast, induced sputum contains many dead and dying cells, making quantitation of levels of inflammatory mediators problematic.

For this reason our study is based on taking respiratory samples from the nose by nasal absorption and curettage. It has long been recognised that there is 'one

For peer review only - http://bmjopen.bmj.com/site/about/guidelines.xhtml

airway', with a strong functional and immunological relationship between the nose and the bronchi (35) (36) (37). Patients with respiratory disease commonly have inflammation of both the airways and nasal passages, with a similar inflammatory infiltrate in the lower and upper airways. It is now possible to obtain repeated samples of nasal exudates before and after nasal challenge in a relatively non-invasive manner by techniques employing strips of nasal synthetic absorptive matrix (SAM) inserted into the nostril in the technique of nasosorption. Our experience with SAM for nasosorption has been published with regard to cytokines and chemokines in children with allergic rhinitis (38), infants with a family history of atopy (39), and in atopic adults after nasal allergen challenge (NAC) (40). In addition, nasal epithelial curettage employing the Rhinoprobe™ device is useful to obtain a pinhead of mucosal tissue, in a technique that does not require local anaesthesia (41). This approach has the advantages of being safe and comfortable for the patient, yet providing high quality samples for transcriptomic analysis.

It is well documented that cigarette smoke is a complex stimulus with a variety of both acute and chronic effects reported in the literature. We set out to design a study to map the acute inflammatory response to smoke in the human respiratory system and circulating cells, with a view to providing a comprehensive molecular signature of smoking–related events in COPD. We believe that our model of cigarette smoke exposure complements the clinical research model of Tam Loi et al. (26). These approaches should lead to advances within the field of assessment of smoking-related immunopathology; therefore we have taken this opportunity to share our rationale and protocol.

Aims of the study

Our study uses non-invasive techniques for sampling, to which 'omic' technology will be utilized for the comprehensive characterization of this complex multi-faceted disease, with the aim of identifying disease-dependent whole system responses to acute cigarette smoke challenge (42). The transcriptomic analysis in this study has been designed to examine changes in gene expression under the specific physiological condition of acute cigarette smoking. This may in future allow for early intervention in populations exhibiting similar gene expression profiles to those observed in established COPD. This approach also provides a diagnostic profile of patients so that treatment can be targeted and personalized. Theoretically, it may be possible to sub-classify COPD populations, who phenotypically appear similar. In addition, there is a need to analyse products of these genes, given that transcriptomic analysis does not solely account for the diversity in protein production and cellular metabolites. A range of cytokines will be measured before and after an acute cigarette challenge to identify biomarkers and cell-signaling pathways associated with COPD. Metabolic profiling will be used to detect the physiological changes induced by a cigarette challenge. Metabolic signatures may provide prognostic, diagnostic and surrogate markers for COPD, and identify simple non-invasive markers of drug responses for future therapies (43). These investigations will be carried out on nasal lining fluid, nasal curettage, and blood, to analyse local and systemic changes over a five hour period following a 2 cigarette challenge. Finally, it has been suggested that stable COPD patients have increased platelet

For peer review only

reactivity including circulating platelet-monocyte aggregates (44), therefore, potential cardiovascular effects will also be assessed by measuring platelet aggregation in these subjects.

Primary objective:

To develop a novel cigarette challenge *in vivo* model incorporating full network biology analysis of transcriptomic, metabolomic and cytokine/chemokine changes in the nose and blood of smokers with Global Initiative for Chronic Obstructive Lung Disease (GOLD, www.goldcopd.org) Stage 2 COPD, healthy smokers with normal lung function and non-smokers, post-cigarette or post-sham/dummy cigarette challenge.

Secondary objectives:

1. To stimulate blood *ex vivo* with IL-1β and PAMPs from smokers with GOLD Stage 2 COPD (16) healthy smokers and non-smokers.
2. To identify molecular biomarkers for COPD patients: to assist in defining novel therapeutic targets, to better stratify phenotypes and to facilitate monitoring of patients.
3. To develop a cigarette smoking challenge model in COPD patients with an aim to utilise this in therapeutic trials of novel therapeutic agents.
4. To carry out platelet aggregometry following stimulation with specific agonists, with the aim of understanding the associated pathophysiology of thrombosis and the pharmacology of respective therapies.

For peer review only

Methods

Study Populations

This is a parallel group study in 3 groups of 12 age, sex, ethnicity, smoking history and BMI matched subjects (Figure 1):

- Group 1: Smokers with moderate COPD (GOLD Stage 2)
- Group 2: Healthy smokers with normal lung function (no evidence of COPD)
- Group 3: Healthy subjects who have never smoked

Smokers will be current smokers, smoking at least 5 cigarettes a day, with a minimum pack year history of 10 years. Current cannabis smokers or smokers with a history of moderate or heavy cannabis use will be excluded from the study. Non-smokers must not have smoked a single cigarette in the 12 months prior to the study, and must have smoked less than 100 cigarettes in their lifetime.

Cigarette Smoke Challenge Procedure

One to three screening visits may be required to complete spirometry (and full lung function with gas transfer for carbon monoxide (TLCO) in smokers), 5 slice High Resolution Computerised Tomography (HRCT) (in smokers only) as well as laboratory safety tests, urinalysis, electrocardiogram and physical examination.

On the Cigarette Challenge Day, all subjects will be required to attend our unit at 9am. All subjects must have fasted and refrained from smoking from 9pm on evening prior to the scheduled challenge. Following baseline investigations, smokers in Groups 1 and 2 will smoke 2 cigarettes back to back, in a controlled environment, exhaling the smoke via their nostrils, whilst non-smokers carry out normal tidal breathing over a 10 minute period (Figure 2).

Schedule of Sampling

All subjects will have nasal epithelial curettage and nasosorption procedures with serial blood samples (Figures 3, 4 and 5). Serial nicotine and cotinine levels will also be taken to plot the relative smoke exposure of each individual, as well as providing an objective measure of their baseline smoking habit and clearance of nicotine from their system. Full blood counts and clotting studies will also be performed during the study to ensure patients have not developed any biochemically relevant illnesses or clotting abnormalities.

Analytical Methods

Nicotine / Cotinine

Nicotine, cotinine and 3-hydroxy-cotinine will be measured in serum over the whole time course. Analysis by capillary gas chromatography will be carried out by Advanced Bioanalytical Service Laboratories (Welwyn Garden City, UK).

8-Isoprostane Enzyme Immuno Assay (EIA)

Measurement of 8-Isoprostane will be carried out in both serum and nasoabsorption fluid at all time points using an 8-isoprostane EIA kit (Cayman Chemicals, Ann Arbor, MI).

For peer review only

Metabolomics

In view of the multiple factors that can influence metabolism, all subjects are required to fast for 12 hours before the challenge begins; during this time they may only consume water. Metabolomic profiling, to be conducted by Metabolon® (North Carolina, USA), measures an extensive range of metabolites (<1000 Da) in plasma and nasal lining fluid after cigarette smoke. Metabolomic analysis will include amino acids, carbohydrates, lipids, nucleic acids and co-factors, molecules of redox homeostasis (e.g. glutathione), organic acids, and small peptides. Importantly, many of the catabolites or biosynthetic intermediates of these metabolites are also detected, assisting in elucidating underlying mechanistic insight. Blood and nasoabsorption samples will be taken at all time points listed in Figure 3.

Transcriptomics

Two nasal epithelial curettage samples will be taken using a Rhinprobe™ pre-challenge and at 300 minutes post-challenge. RNA will be extracted using TRIzol® (Invitrogen, Paisley, UK). Blood will be taken at pre-challenge, 20 and 300 minutes post-challenge with RNA extracted using the PAXgene Blood RNA extraction kit (PreAnalytiX GmbH, Hombrechtikon, Switzerland). RNA will be run on an Illumina HT12 v4 (RefSeq Build 38 Rel 22) chip (Illumina, San Diego, USA) with analysis carried out using a dedicated array analysis programme GeneSpring GX 11.3 of all genes. Genes which have changed significantly will be identified using unpaired t-tests ($p<0.05$) with Benjamini-Hochberg False Discovery Rate (FDR) correction. Genes will be reported as 1.2, 1.5 and 2 fold increases. Quality and assessment of global transcriptome changes will be assessed using principle component analysis in GeneSpring. Genes that have changed significantly will also be further explored using a dedicated pathway analysis tool (Ingenuity Systems Pathway Analysis software [IPA]) techniques.

Ex-vivo Peripheral Whole Blood Stimulation

Stimulation of whole blood taken pre-challenge and at 20 and 300 minutes post-challenge will be performed using a range of toll like receptor (TLR) agonists to obtain a 24 hour dose response curve of the following ligands: LPS (TLR4), FSL-1 (TLR6/2), Pam3CSK4 (TLR1/2), Poly(I:C) (TLR3) and IL-1β (Invivogen, San Diego, USA). Serum will then be removed and subsequently measured for CXCL8 and IL-1β by ELISA (R&D Systems, Abingdon, UK).

Platelet aggregation

For platelet aggregation studies, blood will be collected pre-challenge and at 20 and 300 minutes post-challenge. Platelet rich plasma (PRP) will be aliquoted into individual wells of half-area 96-well plates coated with gelatin and one of seven concentrations of arachidonic acid (AA), adenosine diphosphate (ADP), collagen, epinephrine (EPI), ristocetin, TRAP-6 amide or U46619. Platelet aggregation will be determined by changes in light absorbance, and release of thromboxane (TX)A2 by ELISA.

Homogeneous Time Resolved Fluorescence (HTRF) Assay

Serum and nasoabsorption fluid will be screened, across all time points, for Prostaglandin E2 and Leukotriene B4 levels using the HTRF assay kits from Cisbio Assays (Bedford, MA).

For peer review only

Chemokine / Cytokine Immunoassay

Using a Meso Scale Discovery (MSD) immunoassay system (MSD®, Maryland, USA), a variety of chemokines, cytokines and vascular markers will be measured in blood and nasoabsorption samples at the time points list above.

Statistical analyses

This is an exploratory clinical study, and we are aware that there may not be detectable differences between groups based on measurement of particular parameters and the size of effects.

For peer review only

Discussion

Clinical challenge models have been fundamental to clinical research in asthma: employing inhalation of agents such as methacholine, histamine, adenosine monophosphate (AMP), allergens and occupational agents such as isocyanate and ozone. In contrast there has been little clinical research on the effects of cigarette smoke *in vivo* involving patient studies, despite this being the known causative agent in COPD. Development of clinical challenge models that involve cigarette smoke thus have relevance to studying respiratory, cardiovascular and neoplastic effects of cigarette smoke.

The major consideration in developing a cigarette smoke challenge model is the ethical aspect of not encouraging a smoker to continue smoking, and ensuring that maximal support is given to the individual to stop smoking. Furthermore, we are studying patients with mild-moderate disease, in whom there may be the possibility of smoking cessation before permanent disability sets in. Other studies have tended to evaluate later stage disease, by which time, smoking cessation has less beneficial effects in terms of lung function (45).

A secondary consideration is ensuring the wellbeing of the scientific and clinical staff involved in the study, and minimizing exposure to cigarette smoke. In our study design, all smokers will be established in the habit and will be actively encouraged to enter into a smoking cessation programme immediately following their cigarette challenge. Our unit has adapted a body plethysmography box, with the addition of a carbon filter and HEPA filter, in order to ensure staff are not exposed to the harmful effects of smoke, If subjects were to receive their cigarette challenge outside of the hospital, there may be confounding effects of additional pollutants, temperature and exercise.

An important feature of the study is that smoking two cigarettes is physiologically relevant as a challenge, and that we were able to document levels of nicotine and metabolites over a 5 hour period. We adopted this aspect of the study having considered the design of pharmacokinetic studies with nicotine delivery devices. We intend to standardize the technique of smoking by giving two Marlboro Red cigarettes, noting the total number of inhalations and encouraging exhalation through the nose. Controlled smoke exposure enables accurate assessment of subjects' smoking exposure by measuring concomitant nicotine and cotinine levels. The majority of studies do not mandate any particular smoking restrictions prior to sampling. In this study it may be possible to formally compare nicotine exposure with levels of induced biomarkers.

A key feature of our acute cigarette smoke challenge model involves fasting and refraining from smoking for 12h before and 5h after having a controlled cigarette smoke exposure. We also ensure that patients are not taking any medication that may interfere with responses. This abstinence is necessary due to the extreme sensitivity of measurements such as metabolomics. Metabolomics involves assessment of levels of small molecules and will include molecules such as dietary constituents and drugs. We take serial blood and nasal samples, in a manner similar to a phase I pharmacokinetic study of exposure to a single dose of drug.

For peer review only

The proposed study involves precision nasal sampling. This is non-invasive, has potential for point–of-care, bedside and clinic monitoring. In contrast, bronchoscopy is a research procedure only undertaken with great care in COPD patients. Hence, non-invasive sampling offers great potential for future use of the model in further observational and drug studies. In contrast to many studies looking at gene expression in cross-sectional populations of smokers, our model has the benefit of acquiring samples longitudinally before and after a challenge with a known trigger of the disease. This increases the power to detect effects of the cigarette smoke challenge. We will thoroughly evaluate the acute response to cigarettes with 10 blood and nasal sampling time points within a 5 hour challenge period.

Given the heterogeneity of the disease and the fact that subjects with significant co-morbidities (such as cardiovascular disease) are excluded, we are likely to be evaluating a sub-population of mild COPD patients. This is inevitable in any COPD study, but worth noting, as it may be that this group behaves differently to those with co-morbidities.

For peer review only

Contribution statement

Clare L. Ross contributed to designing the clinical aspect of the study and writing the manuscript. Neil Galloway-Phillipps contributed to the ethics submission and design of laboratory-based methods for the protocol. Paul C. Armstrong contributed to the design of the platelet protocols, ethics submission and writing the manuscript. Jane A. Mitchell contributed to the writing of the manuscript and design of the overall study. Timothy D. Warner contributed to the design of the platelet protocols and ethics submission. Christopher Brearley contributed to writing of the ethics, over all protocol design and its compliance with GCP. Mari Ito contributed to designing the over all protocol, ethics submission and writing the paper. Sarah Elkin contributed to the design of the clinical protocol and ethics submission. Onn Min Kon contributed to the design of the clinical protocol and ethics submission. Trevor T. Hansel contributed to the design of the overall protocol, ethics submission and writing the manuscript. Mark J. Paul-Clark contributed to the design of the overall protocol, design of the assays, ethics submission and writing the manuscript.

Competing interests

None of the authors that have contributed to this publication have competing interests.

Funding

This study was funded by the Wellcome Trust and Dainippon Sumitomo Pharma Co Ltd, Osaka, Japan.

For peer review only

Reference List

(1) Thun MJ, Carter BD, Feskanich D, Freedman ND, Prentice R, Lopez AD et al. 50-year trends in smoking-related mortality in the United States. N Engl J Med 2013 January 24;368(4):351-64.

(2) Decramer M, Janssens W. Chronic obstructive pulmonary disease and comorbidities. Lancet Respir Med 2013 March;1(1):73-83.

(3) Strulovici-Barel Y, Omberg L, O'Mahony M, Gordon C, Hollmann C, Tilley AE et al. Threshold of biologic responses of the small airway epithelium to low levels of tobacco smoke. Am J Respir Crit Care Med 2010 December 15;182(12):1524-32.

(4) van d, V, Postma DS, Timens W, ten Hacken NH. Acute effects of cigarette smoke on inflammation and oxidative stress: a review. Thorax 2004 August;59(8):713-21.

(5) Lokke A, Lange P, Scharling H, Fabricius P, Vestbo J. Developing COPD: a 25 year follow up study of the general population. Thorax 2006 November;61(11):935-9.

(6) Rennard SI, Vestbo J. COPD: the dangerous underestimate of 15%. Lancet 2006 April 15;367(9518):1216-9.

(7) McDonough JE, Yuan R, Suzuki M, Seyednejad N, Elliott WM, Sanchez PG et al. Small-airway obstruction and emphysema in chronic obstructive pulmonary disease. N Engl J Med 2011 October 27;365(17):1567-75.

(8) Brusselle GG, Joos GF, Bracke KR. New insights into the immunology of chronic obstructive pulmonary disease. Lancet 2011 September 10;378(9795):1015-26.

(9) Churg A, Wright JL. Testing drugs in animal models of cigarette smoke-induced chronic obstructive pulmonary disease. Proc Am Thorac Soc 2009 September 15;6(6):550-2.

(10) Walters MJ, Paul-Clark MJ, McMaster SK, Ito K, Adcock IM, Mitchell JA. Cigarette smoke activates human monocytes by an oxidant-AP-1 signaling pathway: implications for steroid resistance. Mol Pharmacol 2005 November;68(5):1343-53.

(11) Oltmanns U, Chung KF, Walters M, John M, Mitchell JA. Cigarette smoke induces IL-8, but inhibits eotaxin and RANTES release from airway smooth muscle. Respir Res 2005;6:74.

(12) Karimi K, Sarir H, Mortaz E, Smit JJ, Hosseini H, de Kimpe SJ et al. Toll-like receptor-4 mediates cigarette smoke-induced cytokine production by human macrophages. Respir Res 2006;7:66.

(13) Birrell MA, Wong S, Catley MC, Belvisi MG. Impact of tobacco-smoke on key signaling pathways in the innate immune response in lung macrophages. J Cell Physiol 2008 January;214(1):27-37.

(14) Wang JH, Kim H, Jang YJ. Cigarette smoke extract enhances rhinovirus-induced toll-like receptor 3 expression and interleukin-8 secretion in A549 cells. Am J Rhinol Allergy 2009 November;23(6):e5-e9.

(15) Pace E, Ferraro M, Siena L, Melis M, Montalbano AM, Johnson M et al. Cigarette smoke increases Toll-like receptor 4 and modifies lipopolysaccharide-mediated responses in airway epithelial cells. Immunology 2008 July;124(3):401-11.

For peer review only

(16) Paul-Clark MJ, Sorrentino R, Bailey LK, Sriskandan S, Mitchell JA. Gram-positive and Gram-negative bacteria synergize with oxidants to release CXCL8 from innate immune cells. Mol Med 2008 May;14(5-6):238-46.
(17) Wright WR, Parzych K, Crawford D, Mein C, Mitchell JA, Paul-Clark MJ. Inflammatory transcriptome profiling of human monocytes exposed acutely to cigarette smoke. PLoS ONE 2012;7(2):e30120.
(18) Marwick JA, Wallis G, Meja K, Kuster B, Bouwmeester T, Chakravarty P et al. Oxidative stress modulates theophylline effects on steroid responsiveness. Biochem Biophys Res Commun 2008 December 19;377(3):797-802.
(19) Silverman EK, Palmer LJ, Mosley JD, Barth M, Senter JM, Brown A et al. Genomewide linkage analysis of quantitative spirometric phenotypes in severe early-onset chronic obstructive pulmonary disease. Am J Hum Genet 2002 May;70(5):1229-39.
(20) Vulimiri SV, Misra M, Hamm JT, Mitchell M, Berger A. Effects of mainstream cigarette smoke on the global metabolome of human lung epithelial cells. Chem Res Toxicol 2009 March 16;22(3):492-503.
(21) Spira A, Beane J, Pinto-Plata V, Kadar A, Liu G, Shah V et al. Gene expression profiling of human lung tissue from smokers with severe emphysema. Am J Respir Cell Mol Biol 2004 December;31(6):601-10.
(22) Spira A, Beane J, Shah V, Liu G, Schembri F, Yang X et al. Effects of cigarette smoke on the human airway epithelial cell transcriptome. Proc Natl Acad Sci U S A 2004 July 6;101(27):10143-8.
(23) Spira A, Schembri F, Beane J, Shah V, Liu G, Brody JS. Impact of cigarette smoke on the normal airway transcriptome. Chest 2004 May;125(5 Suppl):115S.
(24) Heguy A, O'Connor TP, Luettich K, Worgall S, Cieciuch A, Harvey BG et al. Gene expression profiling of human alveolar macrophages of phenotypically normal smokers and nonsmokers reveals a previously unrecognized subset of genes modulated by cigarette smoking. J Mol Med (Berl) 2006 April;84(4):318-28.
(25) Halappanavar S, Russell M, Stampfli MR, Williams A, Yauk CL. Induction of the interleukin 6/ signal transducer and activator of transcription pathway in the lungs of mice sub-chronically exposed to mainstream tobacco smoke. BMC Med Genomics 2009;2:56.
(26) Lo Tam Loi AT, Hoonhorst SJ, Franciosi L, Bischoff R, Hoffmann RF, Heijink I et al. Acute and chronic inflammatory responses induced by smoking in individuals susceptible and non-susceptible to development of COPD: from specific disease phenotyping towards novel therapy. Protocol of a cross-sectional study. BMJ Open 2013;3(2).
(27) Schadt EE. Molecular networks as sensors and drivers of common human diseases. Nature 2009 September 10;461(7261):218-23.
(28) Woodruff PG. Novel outcomes and end points: biomarkers in chronic obstructive pulmonary disease clinical trials. Proc Am Thorac Soc 2011 August;8(4):350-5.
(29) Verrills NM, Irwin JA, He XY, Wood LG, Powell H, Simpson JL et al. Identification of novel diagnostic biomarkers for asthma and chronic obstructive pulmonary disease. Am J Respir Crit Care Med 2011 June 15;183(12):1633-43.

For peer review only

(30) Han MK, Agusti A, Calverley PM, Celli BR, Criner G, Curtis JL et al. Chronic obstructive pulmonary disease phenotypes: the future of COPD. Am J Respir Crit Care Med 2010 September 1;182(5):598-604.

(31) Vestbo J, Anderson W, Coxson HO, Crim C, Dawber F, Edwards L et al. Evaluation of COPD Longitudinally to Identify Predictive Surrogate End-points (ECLIPSE). Eur Respir J 2008 April;31(4):869-73.

(32) Singh D, Edwards L, Tal-Singer R, Rennard S. Sputum neutrophils as a biomarker in COPD: findings from the ECLIPSE study. Respir Res 2010;11:77.

(33) MacNee W, Rennard SI, Hunt JF, Edwards LD, Miller BE, Locantore NW et al. Evaluation of exhaled breath condensate pH as a biomarker for COPD. Respir Med 2011 July;105(7):1037-45.

(34) Hillas G, Loukides S, Kostikas K, Bakakos P. Biomarkers obtained by non-invasive methods in patients with COPD: where do we stand, what do we expect? Curr Med Chem 2009;16(22):2824-38.

(35) Chanez P, Vignola AM, Vic P, Guddo F, Bonsignore G, Godard P et al. Comparison between nasal and bronchial inflammation in asthmatic and control subjects. Am J Respir Crit Care Med 1999 February;159(2):588-95.

(36) American Thoracic Society Workshop. Immunobiology of asthma and rhinitis. Pathogenic factors and therapeutic options. Am J Respir Crit Care Med 1999 November;160(5 Pt 1):1778-87.

(37) Gelfand EW. Inflammatory mediators in allergic rhinitis. J Allergy Clin Immunol 2004 November;114(5 Suppl):S135-S138.

(38) Chawes BLK, Edwards MJ, Shamji B, Walker C, Nicholson GC, Tan AJ et al. A letter to the editor: A novel method for assessing unchallenged levels of mediators in nasal epithelial lining fluid. J Allergy Clin Immunol 2010;125:1387-89.

(39) Folsgaard NV, Chawes BL, Rasmussen MA, Bischoff AL, Carson CG, Stokholm J et al. Maternal atopic skewing of the neonatal nasal cytokine signature. Am J Resp Crit Care Med 2011.

(40) Nicholson GC, Kariyawasam HH, Tan AJ, Hohlfeld JM, Quinn D, Walker C et al. The effects of an anti-IL-13 mAb on cytokine levels and nasal symptoms following nasal allergen challenge. J Allergy Clin Immunol 2011 October;128(4):800-7.

(41) Nathan RA, Eccles R, Howarth PH, Steinsvag SK, Togias A. Objective monitoring of nasal patency and nasal physiology in rhinitis. J Allergy Clin Immunol 2005 March;115(3 Suppl 1):S442-S459.

(42) Schadt EE, Friend SH, Shaywitz DA. A network view of disease and compound screening. Nat Rev Drug Discov 2009 April;8(4):286-95.

(43) Nicholson JK, Lindon JC. Systems biology: Metabonomics. Nature 2008 October 23;455(7216):1054-6.

(44) Maclay JD, McAllister DA, Johnston S, Raftis J, McGuinnes C, Deans A et al. Increased platelet activation in patients with stable and acute exacerbation of COPD. Thorax 2011 September;66(9):769-74.

(45) Scanlon PD, Connett JE, Waller LA, Altose MD, Bailey WC, Sonia BA et al. Smoking cessation and lung function in mild-to-moderate chronic obstructive pulmonary disease. The lung health study. Am J Respir Crit Care Med 2000 February 1;161(2 Pt 1):381-90.

For peer review only

Figure Footnotes

Figure 1. Cigarette Smoke Challenge Model: Inclusion Criteria

Figure 2: Cigarette Smoke Challenge Model

HEPA= high efficiency particulate absorption

Figure 3: Schedule of Assessments

SAM=synthetic absorptive matrix, TLR=Toll-like receptor

Figure 4: Nasal Sampling Methods

Figure 5. Assessment Parameters for Cigarette Smoke Exposure and the Nasal and Systemic Immune Response

For peer review only

Fig. 1. Cigarette Smoke Challenge Model: Inclusion Criteria

General criteria for 3 group of subjects, each of 12 individuals
45 to 75 years old
Good general health, with no chronic illnesses
No prescribed anti-inflammatory medications (including statins)
Females of childbearing potential have a negative pregnancy test

Group 1: Smokers with GOLD Stage 2 COPD
Post-bronchodilator FEV_1 50-79%; Forced Expiratory Ratio <70%
TLCO of <80% of normal
Emphysema on 5 slice HRCT scan of chest

Smokers:
Current: ≥5 cigs/day
History: ≥10 pack yrs

Group 2: Healthy Smokers
Post-bronchodilator FEV_1 ≥80%; Forced Expiratory Ratio ≥ 70%
TLCO ≥ 80% of normal
Normal 5 slice HRCT scan of chest

Group 3: Healthy Non-Smokers
Post-bronchodilator FEV_1 ≥80%; Forced Expiratory Ratio ≥ 70%
TLCO & HRCT not done

Non-Smokers:
Have never smoked

For peer review only - http://bmjopen.bmj.com/site/about/guidelines.xhtml

Fig. 2. Cigarette Smoke Challenge Model

All subjects are asked to refrain from smoking for 12h before and 5h after cigarette challenge.
All subjects are fasted and permitted to drink water for 12h before and 5h after cigarette challenge.

Groups 1 & 2: Smokers
On the morning of the challenge, following baseline investigations, each subject is asked to smoke 2 Marlboro Red cigarettes, one after the other, over a 10 min period in a ventilated chamber. They inhale normally, but exhale only through the nose.

Groups 3: Non-Smokers
On the morning of the challenge, following baseline investigations, each subject is asked to breath normally for 10 min in a smoke-free environment.

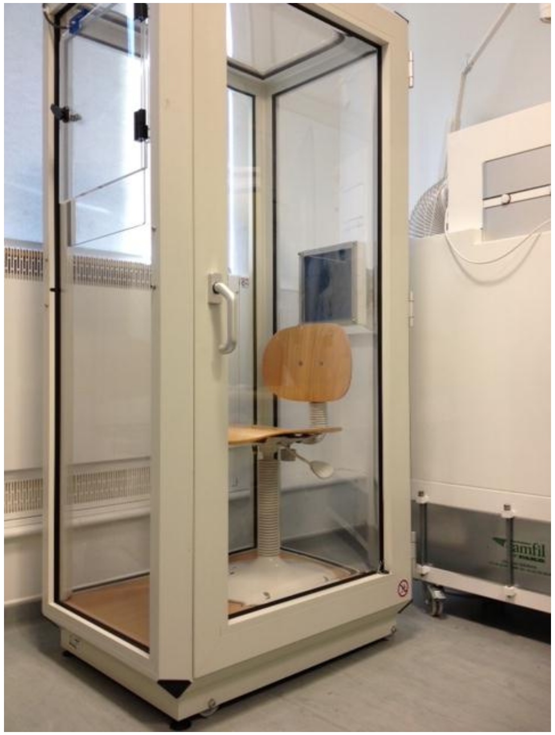

Ventilated chamber with carbon filter attached to ventilation unit in addition to HEPA filter

Fig. 3. Schedule of Assessments

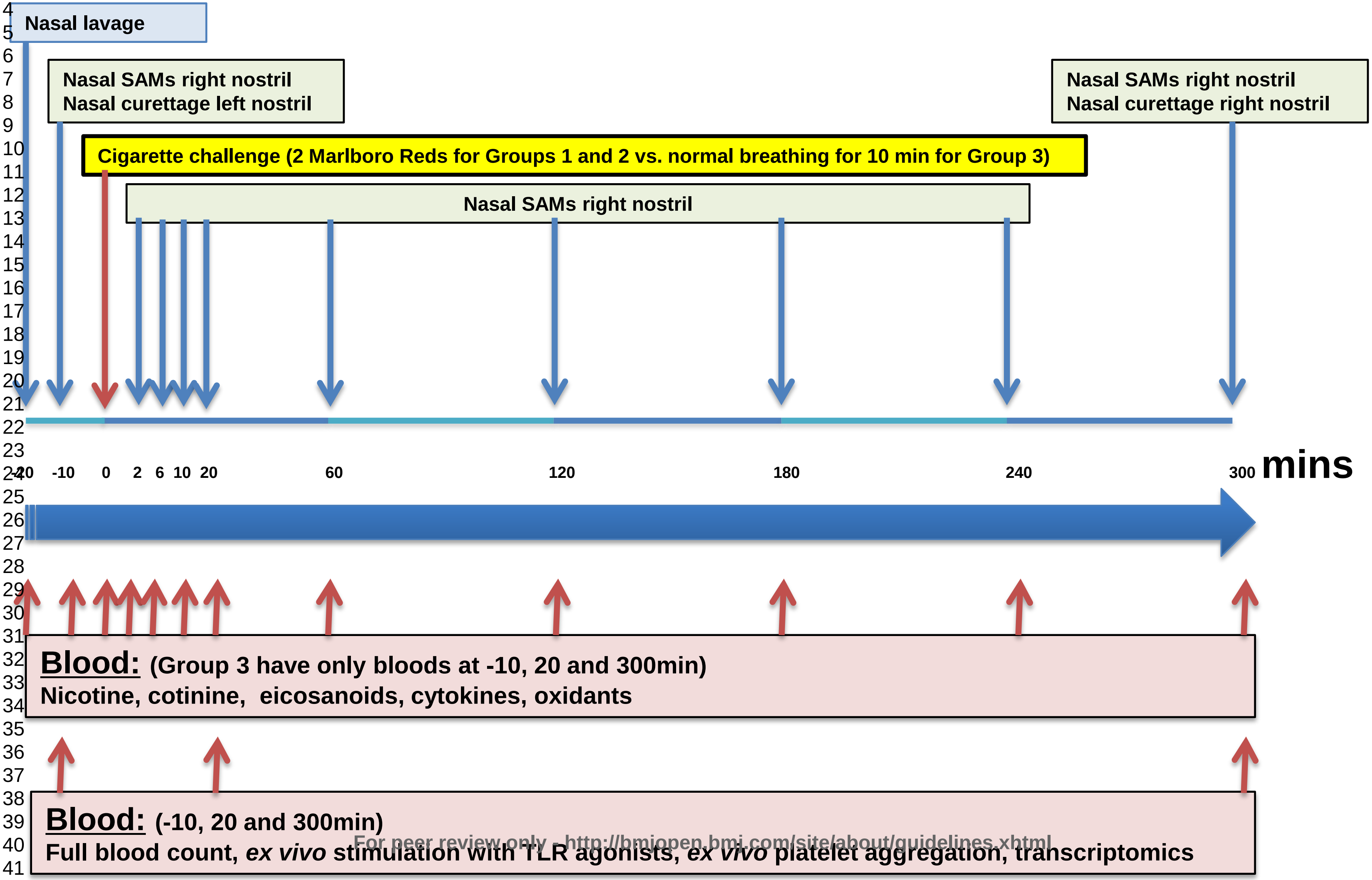

For peer review only - http://bmjopen.bmj.com/site/about/guidelines.xhtml

Fig. 4. Nasal Sampling Methods

Nasosorption using SAM

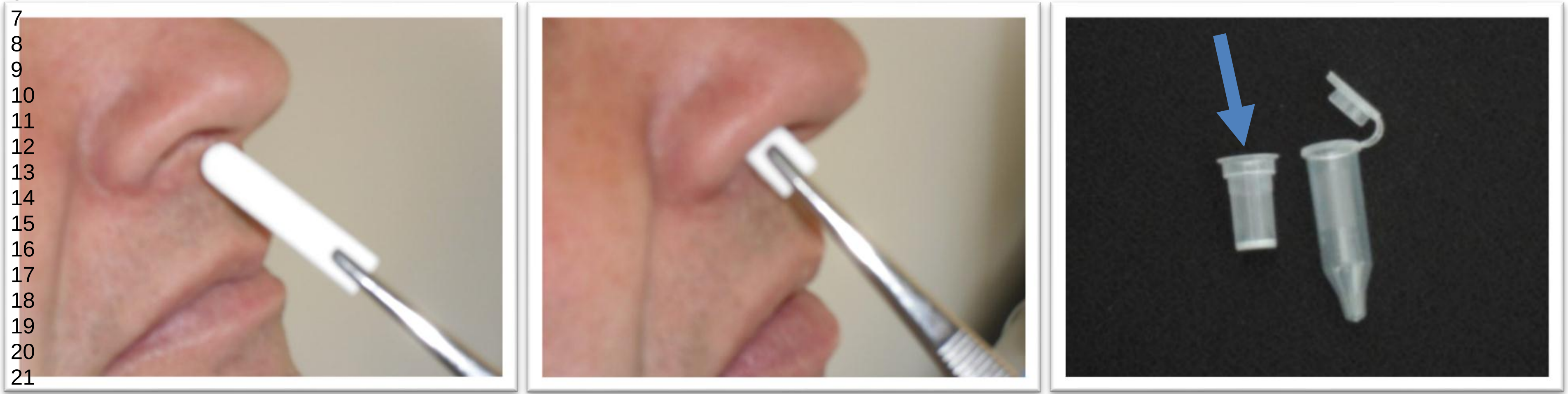

Nasal curettage using a Rhino-probe

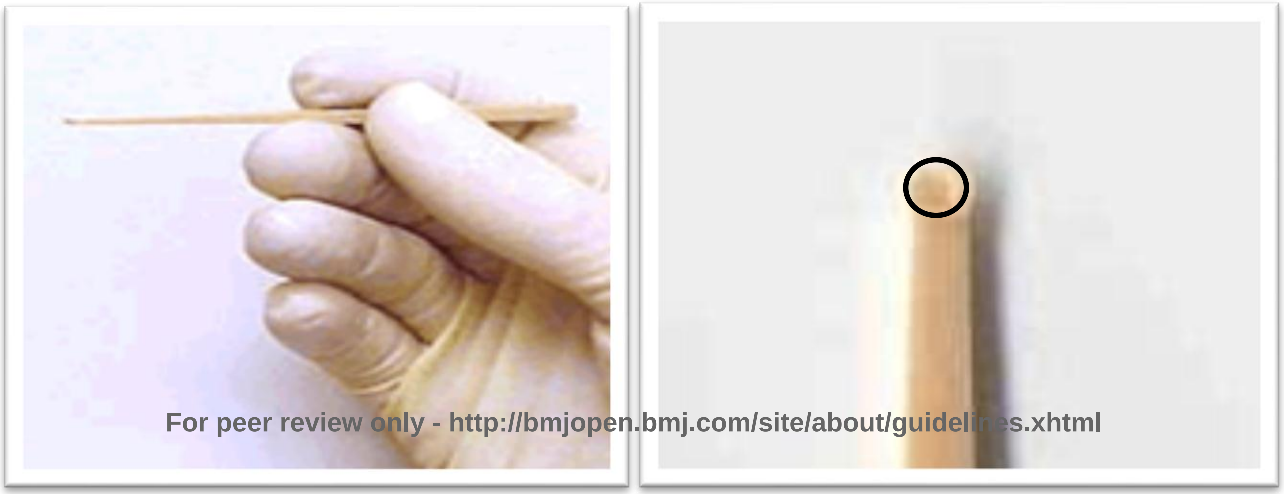

For peer review only - http://bmjopen.bmj.com/site/about/guidelines.xhtml

Fig. 5. Assessment Parameters for Cigarette Smoke Exposure and the Nasal and Systemic Immune Response

Nasosorption (SAM)
- prostanoids: LTB4, LTC4, PGD2
- metabolites
- cytokines and chemokines

Nasal curette transcriptomics

Cigarette smoke correlates of exposure

Plasma:
- nicotine
- cotinine
- 3-hydroxycotinine

Full blood count and coagulation studies

Plasma mediators
- prostanoids: LTB4, LTC4, PGD2
- metabolites
- cytokines and chemokines

Whole blood transcriptomics

Whole blood *ex vivo:*
- TLR-agonist stimulation of leukocytes
- platelet aggregation

Protocol for a human in vivo model of acute cigarette smoke inhalation challenge in smokers with COPD: monitoring the nasal and systemic immune response using a network biology approach

Journal:	*BMJ Open*
Manuscript ID:	bmjopen-2014-005750.R1
Article Type:	Protocol
Date Submitted by the Author:	21-Aug-2014
Complete List of Authors:	Ross, Clare; Imperial College Healthcare NHS Trust, Chest and Allergy<br>Galloway-Phillipps, Neil; Imperial College London, Cardiothoracic Pharmocology<br>Armstrong, Paul; Queen Mary's School of Medicine and Dentistry, William Harvey Research Institute<br>Mitchell, Jane; Imperial College London, National Heart and Lung Institute<br>Warner, Timothy; Queen Mary's School of Medicine and Dentistry, William Harvey Research Institute<br>Brearley, Christopher; Sunovion Pharmaceuticals Europe Ltd,<br>Ito, Mari; Dainippon Sumitomo Pharma Co Ltd, ; Tokyo Medical University, Molecular Regulation for intractable Diseases<br>Tunstall, Tanushree; Imperial College Healthcare NHS Trust, St Mary's Hospital, Clinical Respiratory Research Unit<br>Elkin, Sarah; Imperial College Healthcare NHS Trust, St Mary's Hospital, Respiratory Infection and Medicine, Imperial Clinical Respiratory Research Unit<br>Kon, Onn Min; Imperial College Healthcare NHS Trust, St Mary's Hospital, Respiratory Infection and Medicine, Imperial Clinical Respiratory Research Unit<br>Hansel, Trevor; Imperial College London, Imperial Clinical Respiratory Research Unit (ICRRU) and Biomedical Research Centre (BMRC)<br>Paul-Clark, Mark; Imperial College London, Cardiothoracic Pharmocology; Imperial College HNS Trust, Imperial Clinical Respiratory Research Unit
**Primary Subject Heading**:	Respiratory medicine
Secondary Subject Heading:	Smoking and tobacco, Research methods, Genetics and genomics
Keywords:	Adult thoracic medicine < THORACIC MEDICINE, Chronic airways disease < THORACIC MEDICINE, Emphysema < THORACIC MEDICINE, Immunology < THORACIC MEDICINE

For peer review only

Protocol for a human *in vivo* model of acute cigarette smoke inhalation challenge in smokers with COPD: monitoring the nasal and systemic immune response using a network biology approach

Authors:

Clare L. Ross[1] (MRCP) clare.ross@imperial.nhs.uk

Neil Galloway-Phillipps[2] (BSc) n.galloway-phillipps@imperial.ac.uk

Paul C. Armstrong[3] (PhD) p.c.armstrong@qmul.ac.uk

Jane A. Mitchell[2] (PhD) j.a.mitchell@imperial.ac.uk

Timothy D. Warner[3] (PhD) t.d.warner@qmul.ac.uk

Christopher Brearley[4] (MRCP) chris.brearley@sunovion.eu

Mari Ito[5, 6] (PhD) ito-m@osaka-ra-clinic.com

Tanushree Tunstall[1] (BSc) tanushree.tunstall@imperial.nhs.uk

Sarah Elkin[1] (FRCP) sarah.elkin@imperial.nhs.uk

Onn Min Kon[1] (FRCP) onn.kon@imperial.nhs.uk

Trevor T. Hansel[1*] (FRCPath, PhD) t.hansel@imperial.ac.uk

Mark J. Paul-Clark[2*] (PhD) m.paul-clark@imperial.ac.uk

[1]Imperial Clinical Respiratory Research Unit (ICRRU) and Biomedical Research Centre (BMRC), Centre for Respiratory Infection (CRI), St Mary's Hospital, Imperial College, London, UK

[2] National Heart and Lung Institute, Imperial College, London, UK

[3] William Harvey Research Institute, Barts and The London, Queen Mary's School of Medicine and Dentistry, London, UK

[4] Sunovion Pharmaceuticals Europe Ltd. (Sunovion Europe)

[5] Dainippon Sumitomo Pharma Co Ltd, Osaka, Japan

[6] Department of Molecular Regulation for intractable Diseases, Institute of Medical Science, Tokyo Medical University

*Contributed equally to this project

For peer review only

Correspondence to:

Dr Trevor T. Hansel, Imperial Clinical Respiratory Research Unit (ICRRU), St Mary's Hospital, Praed Street, Paddington, London W2 INY, UK

Tel: +44 203 312 5733; email: t.hansel@imperial.ac.uk

Key Words

COPD, human challenge model, cigarette smoke, inflammation, metabolomics, transcriptomics, nicotine, cytokine, network biology, systems biology

Supported by

Dainippon Sumitomo Pharma Co Ltd, Osaka, Japan National Institute of Healthcare Research (Grant No: R3101002)

NIHR Imperial Biomedical Research Centre (NIHR BMRC)

Imperial Academic Health Science Centre (AHSC)

Imperial Centre for Respiratory Infection (CRI, Grant No: 083567/Z/07/Z)

Wellcome Trust (Grant No: 083429/Z/07/Z)

For peer review only

ABSTRACT

Introduction: Cigarette smoke contributes to a diverse range of diseases including chronic obstructive pulmonary disease (COPD), cardiovascular disorders and many cancers. There currently is a need for human challenge models, to assess the acute effects of a controlled cigarette smoke stimulus, followed by serial sampling of blood and respiratory tissue for advanced molecular profiling. We employ precision sampling of nasal mucosal lining fluid by absorption to allow for the measurement of soluble mediators in eluate. Serial nasal curettage was used for transcriptomic analysis of mucosal tissue.

Methods and analysis: 3 groups of strictly defined subjects will be studied: 12 smokers with COPD (GOLD stage II) with emphysema, 12 matched smokers with normal lung function and no evidence of emphysema, and 12 matched never smokers with normal spirometry. Subjects in the smoking groups are current smokers, and will be given full support to stop smoking immediately after this study. In giving a controlled cigarette smoke stimulus, all subjects will have abstained from smoking for 12 hours, and will smoke 2 cigarettes with expiration through the nose in a ventilated chamber. Before and after inhalation of cigarette smoke, a series of samples will be taken from the blood, nasal mucosal lining fluid, and nasal tissue by curettage. Analysis of plasma nicotine and metabolites in relation to levels of soluble inflammatory mediators in nasal lining fluid and blood, as well as assessing nasal transcriptomics, *ex vivo* blood platelet aggregation and leukocyte responses to TLR-agonists will be undertaken.

Implications: Development of acute cigarette smoke challenge models has promise for the study of molecular effects of smoking in a range of pathological processes.

Ethics and dissemination: This study was approved by the West London National Research Ethics Committee (12/LO/1101). The study findings will be presented at conferences and will be reported in peer-reviewed journals.

For peer review only

ARTICLE SUMMARY

Article focus:

This study examines the acute effects of cigarette smoke in a carefully selected group of smokers and matched controls, employing a network biology approach utilising non-invasive sampling techniques from the upper respiratory tract in conjunction with serial blood sampling.

Key messages:

Smokers with GOLD Stage II COPD with emphysema on HRCT (aged 45-75) are matched with 'healthy' smokers (with normal lung function and imaging) and non-smoking controls.

A controlled stimulus from smoking 2 cigarettes (with nasal exhalation) will take place in a ventilated chamber following a 12 hour abstinence. There will be subsequent serial nasal and blood sampling over a 5 hour period, during which the subject will refrain from further cigarettes.

Strengths and limitations of the study:

This model involves limited numbers of highly selected individuals, and as such selection bias will inevitably occur. However, in conjunction with serial sampling in controlled conditions, this enables discrimination of relatively small changes in levels of cigarette smoke constituents and the subsequent inflammatory response.

It is possible to study acute effects of cigarette smoke in a variety of smoking-related diseases. In this way it may be possible to gain molecular insight into the pathology of COPD, cardiovascular disease and neoplasia.

For peer review only

BACKGROUND

There are profound causative and contributory effects of chronic cigarette smoking in a range of disease processes, as well as considerable mortality (1;2). In order to understand the changes in the lungs due to chronic smoking, it is relevant to assess the immunological responses to acute smoke exposure. Repetitive acute effects of cigarette smoke in susceptible individuals may lead to cumulative irreversible damage (3). Despite cigarette smoking being the most important risk factor for the development of COPD and a major contributory influence in the development of cardiovascular disease, the use of human cigarette challenge models has been under utilised in the investigation of immune and local influences on the disease (4). This is somewhat surprising, as we still know little about the mechanisms involved in smoke-induced inflammation in man.

Whilst several downstream effects of cigarette smoking are common to all smokers, such as anti-oxidant gene activation and aryl hydrocarbon signaling, it is estimated that only 13-50% of smokers actually develop COPD (5) (6), and within this group, there is great clinical heterogeneity (2). The immunopathology of COPD is complex and variable, involving the large airways (bronchitis), small airways (bronchiolitis), lung interstitium (emphysema and interstitial lung disease), pulmonary vasculature (pulmonary artery hypertension) and systemic and cardiovascular complications (7). There is also the added complexity attributable to the innate immune response to oxidants and microbes (8) (9). Our group and others have previously demonstrated that cigarette smoke (extract) can activate human immune and respiratory epithelial cells *in vitro* leading to the release of the pro-inflammatory chemokine CXCL8 (10) (11) (12) (13) (14) (15). In addition, following smoking, blood of smokers is 'primed' to activation *ex vivo* by pathogen associated molecular patterns (PAMPs) including LPS (16). Most recently we have performed a pilot transcriptomic study using human monocytes stimulated *in vitro* and shown that smoke activates and inhibits discrete groups of genes involved in oxidant stress and inflammation (17). In addition there have been individual genomic, transcriptomic and metabolomic studies in cell based (18) (19) (20) (21) (22) (23) (24) and animal models (25) of smoking which have further defined the role of cigarette smoke as an inflammatory insult. However, there is now a need for a multi-systems-based approach in man *in vivo* to truly advance our understanding of how cigarette smoking induces inflammation (26).

A protocol for a study of the effects of smoking in patients with chronic obstructive pulmonary disease (COPD) has recently been reported by Tam Loi and colleagues from Utrecht, Netherlands (27). This group proposes the assessment of acute effects of smoking at 5 minutes after smoking 3 cigarettes, at 2 hours, 24 hours and after a 6 week interval. They also propose assessment of cross-sectional inflammatory responses in different patient groups. In this study sampling from patients consists of blood, sputum and exhaled breath condensate (EBC); and they employ endobronchial sampling for biopsy, epithelial lining fluid and epithelial brushings.

Our group is currently conducting an *in vivo* model of acute cigarette smoke inhalation challenge in smokers with COPD and appropriate controls. Our study differs in selection of patients, and having more defined conditions for cigarette smoke exposure. In addition, we have an intensive sampling schedule over the 5 hours following a controlled cigarette smoke stimulus, with a focus on blood and

For peer review only

nasal non-invasive sampling, during which we assess levels of nicotine and metabolites in relation to pro-inflammatory effects. Such a study has thus far been difficult since access to human airway tissue and secretion samples in a minimally-invasive serial manner has not previously been possible.

There has been recent progress in finding novel biomarkers for COPD (28;29), and a focus on recognizing new phenotypes of COPD (30). GlaxoSmithKline has completed a 3 year longitudinal study in 2180 COPD subjects entitled ECLIPSE (Evaluation of COPD Longitudinally to Identify Predictive Surrogate Endpoints) (31). In terms of the natural history of COPD it was found that sputum neutrophil counts (32) and exhaled breath condensate (EBC) pH was not useful (33). EBC has limitations of dilution and salivary contamination (34). In contrast, induced sputum contains many dead and dying cells, making quantitation of levels of inflammatory mediators problematic.

The nasal epithelium is the first point in the respiratory system where cigarette smoke has contact with the respiratory mucosa. As part of “the one airway concept” that is well established in asthma, there is also considerable evidence for nasal involvement in COPD (35;36). Patients with COPD have chronic nasal symptoms and impaired quality of life (37-39), with upper and lower airway inflammation (40), and exacerbation of COPD is associated with increased pan-airway inflammation(41). In addition, young “healthy smokers” have functional and inflammatory changes in the nose and lower airways (42).

For this reason our study is based on taking respiratory samples from the nose by nasal absorption and curettage. It has long been recognised that there is ‘one airway’, with a strong functional and immunological relationship between the nose and the bronchi (43) (44) (45). Patients with respiratory disease commonly have inflammation of both the airways and nasal passages, with a similar inflammatory infiltrate in the lower and upper airways. It is now possible to obtain repeated samples of nasal exudates before and after nasal challenge in a relatively non-invasive manner by techniques employing strips of nasal synthetic absorptive matrix (SAM) inserted into the nostril in the technique of nasosorption. Our experience with SAM for nasosorption has been published with regard to cytokines and chemokines in children with allergic rhinitis (46), infants with a family history of atopy (47), and in atopic adults after nasal allergen challenge (NAC) (48). In addition, nasal epithelial curettage employing the Rhinoprobe™ device is useful to obtain a pinhead of mucosal tissue, in a technique that does not require local anaesthesia (49). This approach has the advantages of being safe and comfortable for the patient, yet providing high quality samples for transcriptomic analysis.

It is well documented that cigarette smoke is a complex stimulus with a variety of both acute and chronic effects reported in the literature. We set out to design a study to map the acute inflammatory response to smoke in the human respiratory system and circulating cells, with a view to providing a comprehensive molecular signature of smoking–related events in COPD. We believe that our model of cigarette smoke exposure complements the clinical research model of Tam Loi et al. (27). These

For peer review only

approaches should lead to advances within the field of assessment of smoking-related immunopathology; therefore we have taken this opportunity to share our rationale and protocol.

Aims of the study

Our study uses non-invasive techniques for sampling, to which 'omic' technology will be utilized for the comprehensive characterization of this complex multi-faceted disease, with the aim of identifying disease-dependent whole system responses to acute cigarette smoke challenge (50). The transcriptomic analysis in this study has been designed to examine changes in gene expression under the specific physiological condition of acute cigarette smoking. This may in future allow for early intervention in populations exhibiting similar gene expression profiles to those observed in established COPD. This approach also provides a diagnostic profile of patients so that treatment can be targeted and personalized. Theoretically, it may be possible to sub-classify COPD populations, who phenotypically appear similar. In addition, there is a need to analyse products of these genes, given that transcriptomic analysis does not solely account for the diversity in protein production and cellular metabolites. A range of cytokines will be measured before and after an acute cigarette challenge to identify biomarkers and cell-signaling pathways associated with COPD. Metabolic profiling will be used to detect the physiological changes induced by a cigarette challenge. Metabolic signatures may provide prognostic, diagnostic and surrogate markers for COPD, and identify simple non-invasive markers of drug responses for future therapies (51). These investigations will be carried out on nasal lining fluid, nasal curettage, and blood, to analyse local and systemic changes over a five hour period following a 2 cigarette challenge. Finally, it has been suggested that stable COPD patients have increased platelet reactivity including circulating platelet-monocyte aggregates (52), therefore, potential cardiovascular effects will also be assessed by measuring platelet aggregation in these subjects.

Primary objective:

To develop a novel cigarette challenge *in vivo* model incorporating full network biology analysis of transcriptomic, metabolomic and cytokine/chemokine changes in the nose and blood of smokers with Global Initiative for Chronic Obstructive Lung Disease (GOLD, www.goldcopd.org) Stage 2 COPD, healthy smokers with normal lung function and non-smokers, post-cigarette or post-sham/dummy cigarette challenge.

Secondary objectives:

1. To stimulate blood *ex vivo* with IL-1β and PAMPs from smokers with GOLD Stage 2 COPD (16) healthy smokers and non-smokers.
2. To identify molecular biomarkers for COPD patients: to assist in defining novel therapeutic targets, to better stratify phenotypes and to facilitate monitoring of patients.

For peer review only

3. To develop a cigarette smoking challenge model in COPD patients with an aim to utilise this in therapeutic trials of novel therapeutic agents.
4. To carry out platelet aggregometry following stimulation with specific agonists, with the aim of understanding the associated pathophysiology of thrombosis and the pharmacology of respective therapies.

For peer review only

Methods

Study Populations

This is a parallel group study in 3 groups of 12 age, sex, ethnicity, smoking history and BMI matched subjects (Table 1):

- Group 1: Smokers with moderate COPD (GOLD Stage 2)
- Group 2: Healthy smokers with normal lung function (no evidence of COPD)
- Group 3: Healthy subjects who have never smoked

Smokers will be current smokers, smoking at least 5 cigarettes a day, with a minimum pack year history of 10 years. Current cannabis smokers or smokers with a history of moderate or heavy cannabis use will be excluded from the study. Non-smokers must not have smoked a single cigarette in the 12 months prior to the study, and must have smoked less than 100 cigarettes in their lifetime.

Cigarette Smoke Challenge Procedure

One to three screening visits may be required to complete spirometry (and full lung function with gas transfer for carbon monoxide (TLCO) in smokers), 5 slice High Resolution Computerised Tomography (HRCT) (in smokers only) as well as laboratory safety tests, urinalysis, electrocardiogram and physical examination.

On the Cigarette Challenge Day, all subjects will be required to attend our unit at 9am. All subjects must have fasted and refrained from smoking from 9pm on evening prior to the scheduled challenge. Following baseline investigations, smokers in Groups 1 and 2 will smoke 2 cigarettes back to back, in a controlled environment, exhaling the smoke via their nostrils, whilst non-smokers carry out normal tidal breathing over a 10 minute period (Figure 1).

Schedule of Sampling

All subjects will have nasal epithelial curettage and nasosorption procedures with serial blood samples (Figures 2 and 3). Serial nicotine and cotinine levels will also be taken to plot the relative smoke exposure of each individual, as well as providing an objective measure of their baseline smoking habit and clearance of nicotine from their system. Full blood counts and clotting studies will also be performed during the study to ensure patients have not developed any biochemically relevant illnesses or clotting abnormalities.

Analytical Methods

A representative range of non-invasive sampling methods with associated analytical parameters is shown in Table 1.

Nicotine / Cotinine

Nicotine, cotinine and 3-hydroxy-cotinine will be measured in serum over the whole time course. Analysis by capillary gas chromatography will be carried out by Advanced Bioanalytical Service Laboratories (Welwyn Garden City, UK).

8-Isoprostane Enzyme Immuno Assay (EIA)

Measurement of 8-Isoprostane will be carried out in both serum and nasoabsorption fluid at all time points using an 8-isoprostane EIA kit (Cayman Chemicals, Ann Arbor, MI).

Metabolomics

In view of the multiple factors that can influence metabolism, all subjects are required to fast for 12 hours before the challenge begins; during this time they may only consume water. Metabolomic profiling, to be conducted by Metabolon® (North Carolina, USA), measures an extensive range of metabolites (<1000 Da) in plasma and nasal lining fluid after cigarette smoke. Metabolomic analysis will include amino acids, carbohydrates, lipids, nucleic acids and co-factors, molecules of redox homeostasis (e.g. glutathione), organic acids, and small peptides. Importantly, many of the catabolites or biosynthetic intermediates of these metabolites are also detected, assisting in elucidating underlying mechanistic insight. Blood and nasoabsorption samples will be taken at time points listed in Figure 2.

Transcriptomics

Two nasal epithelial curettage samples will be taken using a Rhinprobe™ pre-challenge and at 300 minutes post-challenge. RNA will be extracted using TRIzol® (Invitrogen, Paisley, UK). Blood will be taken at pre-challenge, 20 and 300 minutes post-challenge with RNA extracted using the PAXgene Blood RNA extraction kit (PreAnalytiX GmbH, Hombrechtikon, Switzerland). RNA will be run on an Illumina HT12 v4 (RefSeq Build 38 Rel 22) chip (Illumina, San Diego, USA) with analysis carried out using a dedicated array analysis programme GeneSpring GX 11.3 of all genes. Genes which have changed significantly will be identified using unpaired t-tests ($p<0.05$) with Benjamini-Hochberg False Discovery Rate (FDR) correction. Genes will be reported as 1.2, 1.5 and 2 fold increases. Quality and assessment of global transcriptome changes will be assessed using principle component analysis in GeneSpring. Genes that have changed significantly will also be further explored using a dedicated pathway analysis tool (Ingenuity Systems Pathway Analysis software [IPA]) techniques.

***Ex-vivo* Peripheral Whole Blood Stimulation**

Stimulation of whole blood taken pre-challenge and at 20 and 300 minutes post-challenge will be performed using a range of toll like receptor (TLR) agonists to obtain a 24 hour dose response curve of the following ligands: LPS (TLR4), FSL-1 (TLR6/2), Pam3CSK4 (TLR1/2), Poly(I:C) (TLR3) and IL-1β (Invivogen, San Diego, USA). Serum will then be removed and subsequently measured for CXCL8 and IL-1β by ELISA (R&D Systems, Abingdon, UK).

Platelet aggregation

For platelet aggregation studies, blood will be collected pre-challenge and at 20 and 300 minutes post-challenge. Platelet rich plasma (PRP) will be aliquoted into individual wells of half-area 96-well plates coated with gelatin and one of seven concentrations of arachidonic acid (AA), adenosine diphosphate (ADP), collagen, epinephrine (EPI), ristocetin, TRAP-6 amide or U46619. Platelet aggregation will be determined by changes in light absorbance, and release of thromboxane (TX)A2 by ELISA.

For peer review only

Homogeneous Time Resolved Fluorescence (HTRF) Assay

Serum and nasoabsorption fluid will be screened, across all time points, for Prostaglandin E2 and Leukotriene B4 levels using the HTRF assay kits from Cisbio Assays (Bedford, MA).

Chemokine / Cytokine Immunoassay

Using a Meso Scale Discovery (MSD) immunoassay system (MSD®, Maryland, USA), a variety of chemokines, cytokines and vascular markers will be measured in blood and nasoabsorption samples at the time points list above.

Statistical analyses

This is an exploratory clinical study, and we are aware that there may not be detectable differences between groups based on measurement of particular parameters and the size of effects.

For peer review only

Discussion

Clinical challenge models have been fundamental to clinical research in asthma: employing inhalation of agents such as methacholine, histamine, adenosine monophosphate (AMP), allergens and occupational agents such as isocyanate and ozone. In contrast there has been little clinical research on the effects of cigarette smoke *in vivo* involving patient studies, despite this being the known causative agent in COPD. Development of clinical challenge models that involve cigarette smoke thus have relevance to studying respiratory, cardiovascular and neoplastic effects of cigarette smoke.

The major consideration in developing a cigarette smoke challenge model is the ethical aspect of not encouraging a smoker to continue smoking, and ensuring that maximal support is given to the individual to stop smoking (Table 2). Furthermore, we are studying patients with mild-moderate disease, in whom there may be the possibility of smoking cessation before permanent disability sets in. Other studies have tended to evaluate later stage disease, by which time smoking cessation has less beneficial effects in terms of lung function (53).

A secondary consideration is ensuring the wellbeing of the scientific and clinical staff involved in the study, and minimizing exposure to cigarette smoke. In our study design, all smokers will be established in the habit and will be actively encouraged to enter into a smoking cessation programme immediately following their cigarette challenge. Our unit has adapted a body plethysmography box, with the addition of a carbon filter and HEPA filter, in order to ensure staff are not exposed to the harmful effects of smoke, If subjects were to receive their cigarette challenge outside of the hospital, there may be confounding effects of additional pollutants, temperature and exercise.

An important feature of the study is that smoking two cigarettes is physiologically relevant as a challenge, and that we were able to document levels of nicotine and metabolites over a 5 hour period. We adopted this aspect of the study having considered the design of pharmacokinetic studies with nicotine delivery devices. We intend to standardize the technique of smoking by giving two Marlboro Red cigarettes, noting the total number of inhalations and encouraging exhalation through the nose. Controlled smoke exposure enables accurate assessment of subjects' smoking exposure by measuring concomitant nicotine and cotinine levels. The majority of studies do not mandate any particular smoking restrictions prior to sampling. In this study it may be possible to formally compare nicotine exposure with levels of induced biomarkers.

A key feature of our acute cigarette smoke challenge model involves fasting and refraining from smoking for 12h before and 5h after having a controlled cigarette smoke exposure. We also ensure that patients are not taking any medication that may interfere with responses. This abstinence is necessary due to the extreme sensitivity of measurements such as metabolomics. Metabolomics involves assessment of levels of small molecules and will include molecules such as dietary constituents and drugs. We take serial blood and nasal samples, in a manner similar to a phase I pharmacokinetic study of exposure to a single dose of drug.

For peer review only

The proposed study involves precision nasal sampling. This is non-invasive, has potential for point–of-care, bedside and clinic monitoring. In contrast, bronchoscopy is a research procedure only undertaken with great care in COPD patients. Hence, non-invasive sampling offers great potential for future use of the model in further observational and drug studies. In contrast to many studies looking at gene expression in cross-sectional populations of smokers, our model has the benefit of acquiring samples longitudinally before and after a challenge with a known trigger of the disease. This increases the power to detect effects of the cigarette smoke challenge. We will thoroughly evaluate the acute response to cigarettes with 10 blood and nasal sampling time points within a 5 hour challenge period.

Given the heterogeneity of the disease and the fact that subjects with significant co-morbidities (such as cardiovascular disease) are excluded, we are likely to be evaluating a sub-population of mild COPD patients. This is inevitable in any COPD study, but worth noting, as it may be that this group behaves differently to those with co-morbidities.

The setting up of such studies, using a fully integrated approach that incorporates network biology, where sampling occurs after the administration of causative factors will help us better understand disease and design more robust clinical trials. This type of approach is illustrated in Figure 4 has a far ranging applications to a number of chronic inflammatory diseases.

For peer review only

Table 1.

Summary of Inclusion Criteria and Assessments in the Acute Cigarette Smoke Challenge

Inclusion Criteria	Sample and Assessment Parameters	References
**General criteria for 3 group of subjects, each of 12 individuals**<br>• 45 to 75 years old<br>• Good general health, with no chronic illnesses<br>• No prescribed anti-inflammatory medications (including statins)<br>• Females of childbearing potential have a negative pregnancy test	**Plasma:**<br>Cigarette smoke correlates of exposure<br>• nicotine<br>• cotinine<br>• 3-hydroxycotinine<br>**Plasma** mediators<br>• prostanoids<br>• metabolites<br>• cytokines & chemokines<br>**Whole blood** *ex vivo:*<br>• transcriptomics<br>• TLR-agonist stimulation of leukocytes<br>• platelet aggregation	Nicotine (54)<br><br>Serum cytokines (29;29;55-59)<br><br>Serum metabolites (60-62)<br><br>Blood *ex-vivo* stimulation (16;17)
**Smokers:**<br>Current: $\geq$5 cigs/day<br>History: $\geq$10 pack yrs<br>**Non-Smokers:**<br>Have never smoked	**Nasosorption (SAM)**<br>• prostanoids: LTB4, LTC4, PGD2<br>• metabolites<br>• cytokines and chemokines	
**Group 1: Smokers with GOLD Stage 2 COPD**<br>Post-bronchodilator FEV_1 50-79%; Forced Expiratory Ratio <70%<br>TLCO of <80% of normal<br>Emphysema on 5 slice HRCT scan chest	**Nasal curette**<br>• immunohistology<br>• transcriptomics<br>• flow cytometry epithelial culture	
**Group 2: Healthy Smokers**<br>Post-bronchodilator FEV_1 ≥80%; Forced Expiratory Ratio ≥ 70%<br>TLCO ≥ 80% of normal<br>Normal 5 slice HRCT scan of chest	**Exhaled breath condensate (EBC)**	EBC (33;63)
**Group 3: Healthy Non-Smokers**<br>Post-bronchodilator FEV_1 ≥80%; Forced Expiratory Ratio ≥ 70%<br>TLCO and HRCT not done	**Sputum**	Sputum (32;64;65)

For peer review only

Table 2.

Features and Ethical issues with the Acute Cigarette Smoke Challenge

Features of the model	Ethical Issues
**Advantages:**<br>• Human model<br>• Acute-on-chronic inflammation<br>• Serial non-invasive sampling<br>• Combined direct measurement of biomarkers and *ex vivo* stimulation<br>• Limited numbers of strictly defined subjects<br>• Compare with *in vivo* animal models	All subjects must be advised to stop smoking, and offered full clinical, psychological and pharmacological support to carry this out.<br><br>There must be no encouragement for the subject to begin or continue smoking.<br><br>Some frail COPD subjects will have difficulty fasting and refraining from cigarettes for the morning.<br><br>Clinical disease detected through the investigations must be fully treated, regardless of participation in the study.
**Disadvantages:**<br>• Difficulty recruiting a small number of highly defined subjects<br>• Need to validate upper versus lower airway inflammation, including tissue biopsies<br>• Signal parameters must reliably change after acute cigarette smoke exposure<br>• Lung function and CT changes may occur after acute smoke exposure	Some frail COPD subjects will have difficulty fasting and refraining from cigarettes for the morning.<br><br>The subject should not be taking any anti-inflammatory or confounding therapy: therapy must not be withheld.

For peer review only

Contribution statement

Clare L. Ross contributed to designing the clinical aspect of the study and writing the manuscript. Neil Galloway-Phillipps contributed to the ethics submission and design of laboratory-based methods for the protocol. Paul C. Armstrong contributed to the design of the platelet protocols, ethics submission and writing the manuscript. Jane A. Mitchell contributed to the writing of the manuscript and design of the overall study. Timothy D. Warner contributed to the design of the platelet protocols and ethics submission. Christopher Brearley contributed to writing of the ethics, over all protocol design and its compliance with GCP. Mari Ito, Sarah Elkin, Onn Min Kon and Tanushree Tunstall contributed to the design of the clinical protocol, ethics submission and manuscript composition. Trevor T. Hansel was main author contributed to the design of the overall protocol, ethics submission. Mark J. Paul-Clark contributed to the design of the overall protocol, design of the assays, ethics submission and writing the manuscript.

Competing interests

None of the authors that have contributed to this publication have competing interests.

Funding

This study was funded by the Wellcome Trust and Dainippon Sumitomo Pharma Co Ltd, Osaka, Japan.

For peer review only

Figure Footnotes

Figure 1: Cigarette Smoke Challenge Model

HEPA= high efficiency particulate air

Figure 2: Schedule of Assessments

SAM=synthetic absorptive matrix, TLR=Toll-like receptor

Figure 3: Nasal Sampling Methods

Figure 4. Human Integrated Iterative Inflammometry

For peer review only

Reference List

(1) Thun MJ, Carter BD, Feskanich D, et al.50-year trends in smoking-related mortality in the United States. N Engl J Med 2013 January 24;368(4):351-64.

(2) Decramer M, Janssens W. Chronic obstructive pulmonary disease and comorbidities. Lancet Respir Med 2013 March;1(1):73-83.

(3) Strulovici-Barel Y, Omberg L, et al. Threshold of biologic responses of the small airway epithelium to low levels of tobacco smoke. Am J Respir Crit Care Med 2010 December 15;182(12):1524-32.

(4) van d, V, Postma DS, Timens W, et al. Acute effects of cigarette smoke on inflammation and oxidative stress: a review. Thorax 2004 August;59(8):713-21.

(5) Lokke A, Lange P, Scharling H, et al. Developing COPD: a 25 year follow up study of the general population. Thorax 2006 November;61(11):935-9.

(6) Rennard SI, Vestbo J. COPD: the dangerous underestimate of 15%. Lancet 2006 April 15;367(9518):1216-9.

(7) McDonough JE, Yuan R, Suzuki M, et al. Small-airway obstruction and emphysema in chronic obstructive pulmonary disease. N Engl J Med 2011 October 27;365(17):1567-75.

(8) Brusselle GG, Joos GF, Bracke KR. New insights into the immunology of chronic obstructive pulmonary disease. Lancet 2011 September 10;378(9795):1015-26.

(9) Churg A, Wright JL. Testing drugs in animal models of cigarette smoke-induced chronic obstructive pulmonary disease. Proc Am Thorac Soc 2009 September 15;6(6):550-2.

(10) Walters MJ, Paul-Clark MJ, McMaster SK, et al.Cigarette smoke activates human monocytes by an oxidant-AP-1 signaling pathway: implications for steroid resistance. Mol Pharmacol 2005 November;68(5):1343-53.

(11) Oltmanns U, Chung KF, Walters M, et al. Cigarette smoke induces IL-8, but inhibits eotaxin and RANTES release from airway smooth muscle. Respir Res 2005;6:74.

(12) Karimi K, Sarir H, Mortaz E, et al. Toll-like receptor-4 mediates cigarette smoke-induced cytokine production by human macrophages. Respir Res 2006;7:66.

(13) Birrell MA, Wong S, Catley MC, et al. Impact of tobacco-smoke on key signaling pathways in the innate immune response in lung macrophages. J Cell Physiol 2008 January;214(1):27-37.

For peer review only

(14) Wang JH, Kim H, Jang YJ. Cigarette smoke extract enhances rhinovirus-induced toll-like receptor 3 expression and interleukin-8 secretion in A549 cells. Am J Rhinol Allergy 2009 November;23(6):e5-e9.

(15) Pace E, Ferraro M, Siena L, et al. Cigarette smoke increases Toll-like receptor 4 and modifies lipopolysaccharide-mediated responses in airway epithelial cells. Immunology 2008 July;124(3):401-11.

(16) Paul-Clark MJ, Sorrentino R, Bailey LK, et al. Gram-positive and Gram-negative bacteria synergize with oxidants to release CXCL8 from innate immune cells. Mol Med 2008 May;14(5-6):238-46.

(17) Wright WR, Parzych K, Crawford D, et al. Inflammatory transcriptome profiling of human monocytes exposed acutely to cigarette smoke. PLoS ONE 2012;7(2):e30120.

(18) Marwick JA, Wallis G, Meja K, et al. Oxidative stress modulates theophylline effects on steroid responsiveness. Biochem Biophys Res Commun 2008 December 19;377(3):797-802.

(19) Silverman EK, Palmer LJ, Mosley JD, et al. Genomewide linkage analysis of quantitative spirometric phenotypes in severe early-onset chronic obstructive pulmonary disease. Am J Hum Genet 2002 May;70(5):1229-39.

(20) Vulimiri SV, Misra M, Hamm JT, et al. Effects of mainstream cigarette smoke on the global metabolome of human lung epithelial cells. Chem Res Toxicol 2009 March 16;22(3):492-503.

(21) Spira A, Beane J, Pinto-Plata V, et al. Gene expression profiling of human lung tissue from smokers with severe emphysema. Am J Respir Cell Mol Biol 2004 December;31(6):601-10.

(22) Spira A, Beane J, Shah V, Liu G, Schembri F, Yang X et al. Effects of cigarette smoke on the human airway epithelial cell transcriptome. Proc Natl Acad Sci U S A 2004 July 6;101(27):10143-8.

(23) Spira A, Schembri F, Beane J, et al. Impact of cigarette smoke on the normal airway transcriptome. Chest 2004 May;125(5 Suppl):115S.

(24) Heguy A, O'Connor TP, Luettich K, et al. Gene expression profiling of human alveolar macrophages of phenotypically normal smokers and nonsmokers reveals a previously unrecognized subset of genes modulated by cigarette smoking. J Mol Med (Berl) 2006 April;84(4):318-28.

(25) Halappanavar S, Russell M, Stampfli MR, et al. Induction of the interleukin 6/ signal transducer and activator of transcription pathway in the lungs of mice sub-chronically exposed to mainstream tobacco smoke. BMC Med Genomics 2009;2:56.

(26) Schadt EE. Molecular networks as sensors and drivers of common human diseases. Nature 2009 September 10;461(7261):218-23.

For peer review only

(27) Lo Tam Loi AT, Hoonhorst SJ, Franciosi L, et al. Acute and chronic inflammatory responses induced by smoking in individuals susceptible and non-susceptible to development of COPD: from specific disease phenotyping towards novel therapy. Protocol of a cross-sectional study. BMJ Open 2013;3(2).

(28) Woodruff PG. Novel outcomes and end points: biomarkers in chronic obstructive pulmonary disease clinical trials. Proc Am Thorac Soc 2011 August;8(4):350-5.

(29) Verrills NM, Irwin JA, He XY, et al. Identification of novel diagnostic biomarkers for asthma and chronic obstructive pulmonary disease. Am J Respir Crit Care Med 2011 June 15;183(12):1633-43.

(30) Han MK, Agusti A, Calverley PM, et al. Chronic obstructive pulmonary disease phenotypes: the future of COPD. Am J Respir Crit Care Med 2010 September 1;182(5):598-604.

(31) Vestbo J, Anderson W, Coxson HO, et al.Evaluation of COPD Longitudinally to Identify Predictive Surrogate End-points (ECLIPSE). Eur Respir J 2008 April;31(4):869-73.

(32) Singh D, Edwards L, Tal-Singer R, et al. Sputum neutrophils as a biomarker in COPD: findings from the ECLIPSE study. Respir Res 2010;11:77.

(33) MacNee W, Rennard SI, Hunt JF, et al.Evaluation of exhaled breath condensate pH as a biomarker for COPD. Respir Med 2011 July;105(7):1037-45.

(34) Hillas G, Loukides S, Kostikas K, et al. Biomarkers obtained by non-invasive methods in patients with COPD: where do we stand, what do we expect? Curr Med Chem 2009;16(22):2824-38.

(35) Hurst JR. Upper airway. 3: Sinonasal involvement in chronic obstructive pulmonary disease. Thorax 2010 January;65(1):85-90.

(36) Hakansson K, Konge L, Thomsen SF, et al. Sinonasal inflammation in COPD: a systematic review. Eur Respir J 2013 November;42(5):1402-11.

(37) Hurst JR, Wilkinson TM, Donaldson GC, et al. Upper airway symptoms and quality of life in chronic obstructive pulmonary disease (COPD). Respir Med 2004 August;98(8):767-70.

(38) Hurst JR, Kuchai R, Michael P, et al. Nasal symptoms, airway obstruction and disease severity in chronic obstructive pulmonary disease. Clin Physiol Funct Imaging 2006 July;26(4):251-6.

(39) Caillaud D, Chanez P, Escamilla R, et al. Association of chronic nasal symptoms with dyspnoea and quality-of-life impairment in chronic obstructive pulmonary disease. Respirology 2014 April;19(3):346-52.

For peer review only

(40) Hurst JR, Wilkinson TM, Perera WR, et al. Relationships among bacteria, upper airway, lower airway, and systemic inflammation in COPD. Chest 2005 April;127(4):1219-26.

(41) Hurst JR, Perera WR, Wilkinson TM, et al. Systemic and upper and lower airway inflammation at exacerbation of chronic obstructive pulmonary disease. Am J Respir Crit Care Med 2006 January 1;173(1):71-8.

(42) Nicola ML, Carvalho HB, Yoshida CT, et al.Young "healthy" smokers have functional and inflammatory changes in the nasal and the lower airways. Chest 2014 May;145(5):998-1005.

(43) Chanez P, Vignola AM, Vic P, et al. Comparison between nasal and bronchial inflammation in asthmatic and control subjects. Am J Respir Crit Care Med 1999 February;159(2):588-95.

(44) American Thoracic Society Workshop. Immunobiology of asthma and rhinitis. Pathogenic factors and therapeutic options. Am J Respir Crit Care Med 1999 November;160(5 Pt 1):1778-87.

(45) Gelfand EW. Inflammatory mediators in allergic rhinitis. J Allergy Clin Immunol 2004 November;114(5 Suppl):S135-S138.

(46) Chawes BLK, Edwards MJ, Shamji B, et al. A letter to the editor: A novel method for assessing unchallenged levels of mediators in nasal epithelial lining fluid. J Allergy Clin Immunol 2010;125:1387-89.

(47) Folsgaard NV, Chawes BL, Rasmussen MA, et al. Maternal atopic skewing of the neonatal nasal cytokine signature. Am J Resp Crit Care Med 2011.

(48) Nicholson GC, Kariyawasam HH, Tan AJ, et al. The effects of an anti-IL-13 mAb on cytokine levels and nasal symptoms following nasal allergen challenge. J Allergy Clin Immunol 2011 October;128(4):800-7.

(49) Nathan RA, Eccles R, Howarth PH, et al. Objective monitoring of nasal patency and nasal physiology in rhinitis. J Allergy Clin Immunol 2005 March;115(3 Suppl 1):S442-S459.

(50) Schadt EE, Friend SH, Shaywitz DA. A network view of disease and compound screening. Nat Rev Drug Discov 2009 April;8(4):286-95.

(51) Nicholson JK, Lindon JC. Systems biology: Metabonomics. Nature 2008 October 23;455(7216):1054-6.

(52) Maclay JD, McAllister DA, Johnston S, et al. Increased platelet activation in patients with stable and acute exacerbation of COPD. Thorax 2011 September;66(9):769-74.

(53) Scanlon PD, Connett JE, Waller LA, et al. Smoking cessation and lung function in mild-to-moderate chronic obstructive pulmonary disease. The lung health study. Am J Respir Crit Care Med 2000 February 1;161(2 Pt 1):381-90.

For peer review only

(54) Benowitz NL, Hukkanen J, Jacob P, III. Nicotine chemistry, metabolism, kinetics and biomarkers. Handb Exp Pharmacol 2009;(192):29-60.

(55) Sin DD, Miller BE, Duvoix A, et al. Serum PARC/CCL-18 concentrations and health outcomes in chronic obstructive pulmonary disease. Am J Respir Crit Care Med 2011 May 1;183(9):1187-92.

(56) Cheng DT, Kim DK, Cockayne DA, et al. Systemic Soluble Receptor for Advanced Glycation Endproducts Is a Biomarker of Emphysema and Associated with AGER Genetic Variants in Patients with Chronic Obstructive Pulmonary Disease. Am J Respir Crit Care Med 2013 October 15;188(8):948-57.

(57) Faner R, Tal-Singer R, Riley JH, et al. Lessons from ECLIPSE: a review of COPD biomarkers. Thorax 2014 July;69(7):666-72.

(58) Celli BR, Locantore N, Yates J, et al. Inflammatory biomarkers improve clinical prediction of mortality in chronic obstructive pulmonary disease. Am J Respir Crit Care Med 2012 May 15;185(10):1065-72.

(59) Kim DK, Cho MH, Hersh CP, et al. Genome-wide association analysis of blood biomarkers in chronic obstructive pulmonary disease. Am J Respir Crit Care Med 2012 December 15;186(12):1238-47.

(60) Muller DC, Degen C, Scherer G, et al.Metabolomics using GC-TOF-MS followed by subsequent GC-FID and HILIC-MS/MS analysis revealed significantly altered fatty acid and phospholipid species profiles in plasma of smokers. J Chromatogr B Analyt Technol Biomed Life Sci 2014 September 1;966:117-26.

(61) Ubhi BK, Cheng KK, Dong J, et al. Targeted metabolomics identifies perturbations in amino acid metabolism that sub-classify patients with COPD. Mol Biosyst 2012 October 30;8(12):3125-33.

(62) Ubhi BK, Riley JH, Shaw PA, et al.Metabolic profiling detects biomarkers of protein degradation in COPD patients. Eur Respir J 2012 August;40(2):345-55.

(63) Fens N, van der Schee MP, Brinkman P, et al. Exhaled breath analysis by electronic nose in airways disease. Established issues and key questions. Clin Exp Allergy 2013 July;43(7):705-15.

(64) Qiu W, Cho MH, Riley JH, et al. Genetics of sputum gene expression in chronic obstructive pulmonary disease. PLoS ONE 2011;6(9):e24395.

(65) Singh D, Fox SM, Tal-Singer R, et al. Induced sputum genes associated with spirometric and radiological disease severity in COPD ex-smokers. Thorax 2011 June;66(6):489-95.

For peer review only

Protocol for a human *in vivo* model of acute cigarette smoke inhalation challenge in smokers with COPD: monitoring the nasal and systemic immune response using a network biology approach

Authors:

Clare L. Ross[1] (MRCP) clare.ross@imperial.nhs.uk

Neil Galloway-Phillipps[2] (BSc) n.galloway-phillipps@imperial.ac.uk

Paul C. Armstrong[3] (PhD) p.c.armstrong@qmul.ac.uk

Jane A. Mitchell[2] (PhD) j.a.mitchell@imperial.ac.uk

Timothy D. Warner[3] (PhD) t.d.warner@qmul.ac.uk

Christopher Brearley[4] (MRCP) chris.brearley@sunovion.eu

Mari Ito[5, 6] (PhD) ito-m@osaka-ra-clinic.com

Tanushree Tunstall[1] (BSc) tanushree.tunstall@imperial.nhs.uk

Sarah Elkin[1] (FRCP) sarah.elkin@imperial.nhs.uk

Onn Min Kon[1] (FRCP) onn.kon@imperial.nhs.uk

Trevor T. Hansel[1*] (FRCPath, PhD) t.hansel@imperial.ac.uk

Mark J. Paul-Clark[2*] (PhD) m.paul-clark@imperial.ac.uk

[1]Imperial Clinical Respiratory Research Unit (ICRRU) and Biomedical Research Centre (BMRC), Centre for Respiratory Infection (CRI), St Mary's Hospital, Imperial College, London, UK

[2] National Heart and Lung Institute, Imperial College, London, UK

[3] William Harvey Research Institute, Barts and The London, Queen Mary's School of Medicine and Dentistry, London, UK

[4] Sunovion Pharmaceuticals Europe Ltd. (Sunovion Europe)

[5] Dainippon Sumitomo Pharma Co Ltd, Osaka, Japan

[6] Department of Molecular Regulation for intractable Diseases, Institute of Medical Science, Tokyo Medical University

*Contributed equally to this project

For peer review only

Correspondence to:

Dr Trevor T. Hansel, Imperial Clinical Respiratory Research Unit (ICRRU), St Mary's Hospital, Praed Street, Paddington, London W2 INY, UK

Tel: +44 203 312 5733; email: t.hansel@imperial.ac.uk

Key Words

COPD, human challenge model, cigarette smoke, inflammation, metabolomics, transcriptomics, nicotine, cytokine, network biology, systems biology

Supported by

Dainippon Sumitomo Pharma Co Ltd, Osaka, Japan National Institute of Healthcare Research (Grant No: R3101002)

NIHR Imperial Biomedical Research Centre (NIHR BMRC)

Imperial Academic Health Science Centre (AHSC)

Imperial Centre for Respiratory Infection (CRI, Grant No: 083567/Z/07/Z)

Wellcome Trust (Grant No: 083429/Z/07/Z)

For peer review only

ABSTRACT

Introduction: Cigarette smoke contributes to a diverse range of diseases including chronic obstructive pulmonary disease (COPD), cardiovascular disorders and many cancers. There currently is a need for human challenge models, to assess the acute effects of a controlled cigarette smoke stimulus, followed by serial sampling of blood and respiratory tissue for advanced molecular profiling. We employ precision sampling of nasal mucosal lining fluid by absorption to allow for the measurement of soluble mediators in eluate. Serial nasal curettage was used for transcriptomic analysis of mucosal tissue.

Methods and analysis: 3 groups of strictly defined subjects will be studied: 12 smokers with COPD (GOLD stage II) with emphysema, 12 matched smokers with normal lung function and no evidence of emphysema, and 12 matched never smokers with normal spirometry. Subjects in the smoking groups are current smokers, and will be given full support to stop smoking immediately after this study. In giving a controlled cigarette smoke stimulus, all subjects will have abstained from smoking for 12 hours, and will smoke 2 cigarettes with expiration through the nose in a ventilated chamber. Before and after inhalation of cigarette smoke, a series of samples will be taken from the blood, nasal mucosal lining fluid, and nasal tissue by curettage. Analysis of plasma nicotine and metabolites in relation to levels of soluble inflammatory mediators in nasal lining fluid and blood, as well as assessing nasal transcriptomics, *ex vivo* blood platelet aggregation and leukocyte responses to TLR-agonists will be undertaken.

Implications: Development of acute cigarette smoke challenge models has promise for the study of molecular effects of smoking in a range of pathological processes.

Ethics and dissemination: This study was approved by the West London National Research Ethics Committee (12/LO/1101). The study findings will be presented at conferences and will be reported in peer-reviewed journals.

For peer review only

ARTICLE SUMMARY

Article focus:

This study examines the acute effects of cigarette smoke in a carefully selected group of smokers and matched controls, employing a network biology approach utilising non-invasive sampling techniques from the upper respiratory tract in conjunction with serial blood sampling.

Key messages:

Smokers with GOLD Stage II COPD with emphysema on HRCT (aged 45-75) are matched with ‘healthy’ smokers (with normal lung function and imaging) and non-smoking controls.

A controlled stimulus from smoking 2 cigarettes (with nasal exhalation) will take place in a ventilated chamber following a 12 hour abstinence. There will be subsequent serial nasal and blood sampling over a 5 hour period, during which the subject will refrain from further cigarettes.

Strengths and limitations of the study:

This model involves limited numbers of highly selected individuals, and as such selection bias will inevitably occur. However, in conjunction with serial sampling in controlled conditions, this enables discrimination of relatively small changes in levels of cigarette smoke constituents and the subsequent inflammatory response.

It is possible to study acute effects of cigarette smoke in a variety of smoking-related diseases. In this way it may be possible to gain molecular insight into the pathology of COPD, cardiovascular disease and neoplasia.

BACKGROUND

There are profound causative and contributory effects of chronic cigarette smoking in a range of disease processes, as well as considerable mortality (1;2). In order to understand the changes in the lungs due to chronic smoking, it is relevant to assess the immunological responses to acute smoke exposure. Repetitive acute effects of cigarette smoke in susceptible individuals may lead to cumulative irreversible damage (3). Despite cigarette smoking being the most important risk factor for the development of COPD and a major contributory influence in the development of cardiovascular disease, the use of human cigarette challenge models has been under utilised in the investigation of immune and local influences on the disease (4). This is somewhat surprising, as we still know little about the mechanisms involved in smoke-induced inflammation in man.

Whilst several downstream effects of cigarette smoking are common to all smokers, such as anti-oxidant gene activation and aryl hydrocarbon signaling, it is estimated that only 13-50% of smokers actually develop COPD (5) (6), and within this group,

For peer review only

there is great clinical heterogeneity (2). The immunopathology of COPD is complex and variable, involving the large airways (bronchitis), small airways (bronchiolitis), lung interstitium (emphysema and interstitial lung disease), pulmonary vasculature (pulmonary artery hypertension) and systemic and cardiovascular complications (7). There is also the added complexity attributable to the innate immune response to oxidants and microbes (8) (9). Our group and others have previously demonstrated that cigarette smoke (extract) can activate human immune and respiratory epithelial cells *in vitro* leading to the release of the pro-inflammatory chemokine CXCL8 (10) (11) (12) (13) (14) (15). In addition, following smoking, blood of smokers is 'primed' to activation *ex vivo* by pathogen associated molecular patterns (PAMPs) including LPS (16). Most recently we have performed a pilot transcriptomic study using human monocytes stimulated *in vitro* and shown that smoke activates and inhibits discrete groups of genes involved in oxidant stress and inflammation (17). In addition there have been individual genomic, transcriptomic and metabolomic studies in cell based (18) (19) (20) (21) (22) (23) (24) and animal models (25) of smoking which have further defined the role of cigarette smoke as an inflammatory insult. However, there is now a need for a multi-systems-based approach in man *in vivo* to truly advance our understanding of how cigarette smoking induces inflammation (26).

A protocol for a study of the effects of smoking in patients with chronic obstructive pulmonary disease (COPD) has recently been reported by Tam Loi and colleagues from Utrecht, Netherlands (27). This group proposes the assessment of acute effects of smoking at 5 minutes after smoking 3 cigarettes, at 2 hours, 24 hours and after a 6 week interval. They also propose assessment of cross-sectional inflammatory responses in different patient groups. In this study sampling from patients consists of blood, sputum and exhaled breath condensate (EBC); and they employ endobronchial sampling for biopsy, epithelial lining fluid and epithelial brushings.

Our group is currently conducting an *in vivo* model of acute cigarette smoke inhalation challenge in smokers with COPD and appropriate controls. Our study differs in selection of patients, and having more defined conditions for cigarette smoke exposure. In addition, we have an intensive sampling schedule over the 5 hours following a controlled cigarette smoke stimulus, with a focus on blood and nasal non-invasive sampling, during which we assess levels of nicotine and metabolites in relation to pro-inflammatory effects. Such a study has thus far been difficult since access to human airway tissue and secretion samples in a minimally-invasive serial manner has not previously been possible.

There has been recent progress in finding novel biomarkers for COPD (28;29), and a focus on recognizing new phenotypes of COPD (30). GlaxoSmithKline has completed a 3 year longitudinal study in 2180 COPD subjects entitled ECLIPSE (Evaluation of COPD Longitudinally to Identify Predictive Surrogate Endpoints) (31). In terms of the natural history of COPD it was found that sputum neutrophil counts (32) and exhaled breath condensate (EBC) pH was not useful (33). EBC has limitations of dilution and salivary contamination (34). In contrast, induced sputum contains many dead and dying cells, making quantitation of levels of inflammatory mediators problematic.

For peer review only

The nasal epithelium is the first point in the respiratory system where cigarette smoke has contact with the respiratory mucosa. As part of “the one airway concept” that is well established in asthma, there is also considerable evidence for nasal involvement in COPD (35;36). Patients with COPD have chronic nasal symptoms and impaired quality of life (37-39), with upper and lower airway inflammation (40), and exacerbation of COPD is associated with increased pan-airway inflammation(41). In addition, young “healthy smokers” have functional and inflammatory changes in the nose and lower airways (42).

For this reason our study is based on taking respiratory samples from the nose by nasal absorption and curettage. It has long been recognised that there is ‘one airway’, with a strong functional and immunological relationship between the nose and the bronchi (43) (44) (45). Patients with respiratory disease commonly have inflammation of both the airways and nasal passages, with a similar inflammatory infiltrate in the lower and upper airways. It is now possible to obtain repeated samples of nasal exudates before and after nasal challenge in a relatively non-invasive manner by techniques employing strips of nasal synthetic absorptive matrix (SAM) inserted into the nostril in the technique of nasosorption. Our experience with SAM for nasosorption has been published with regard to cytokines and chemokines in children with allergic rhinitis (46), infants with a family history of atopy (47), and in atopic adults after nasal allergen challenge (NAC) (48). In addition, nasal epithelial curettage employing the Rhinoprobe™ device is useful to obtain a pinhead of mucosal tissue, in a technique that does not require local anaesthesia (49). This approach has the advantages of being safe and comfortable for the patient, yet providing high quality samples for transcriptomic analysis.

It is well documented that cigarette smoke is a complex stimulus with a variety of both acute and chronic effects reported in the literature. We set out to design a study to map the acute inflammatory response to smoke in the human respiratory system and circulating cells, with a view to providing a comprehensive molecular signature of smoking–related events in COPD. We believe that our model of cigarette smoke exposure complements the clinical research model of Tam Loi et al. (27). These approaches should lead to advances within the field of assessment of smoking-related immunopathology; therefore we have taken this opportunity to share our rationale and protocol.

Aims of the study

Our study uses non-invasive techniques for sampling, to which ‘omic’ technology will be utilized for the comprehensive characterization of this complex multi-faceted disease, with the aim of identifying disease-dependent whole system responses to acute cigarette smoke challenge (50). The transcriptomic analysis in this study has been designed to examine changes in gene expression under the specific physiological condition of acute cigarette smoking. This may in future allow for early intervention in populations exhibiting similar gene expression profiles to those observed in established COPD. This approach also provides a diagnostic profile of

For peer review only

patients so that treatment can be targeted and personalized. Theoretically, it may be possible to sub-classify COPD populations, who phenotypically appear similar. In addition, there is a need to analyse products of these genes, given that transcriptomic analysis does not solely account for the diversity in protein production and cellular metabolites. A range of cytokines will be measured before and after an acute cigarette challenge to identify biomarkers and cell-signaling pathways associated with COPD. Metabolic profiling will be used to detect the physiological changes induced by a cigarette challenge. Metabolic signatures may provide prognostic, diagnostic and surrogate markers for COPD, and identify simple non-invasive markers of drug responses for future therapies (51). These investigations will be carried out on nasal lining fluid, nasal curettage, and blood, to analyse local and systemic changes over a five hour period following a 2 cigarette challenge. Finally, it has been suggested that stable COPD patients have increased platelet reactivity including circulating platelet-monocyte aggregates (52), therefore, potential cardiovascular effects will also be assessed by measuring platelet aggregation in these subjects.

Primary objective:

To develop a novel cigarette challenge *in vivo* model incorporating full network biology analysis of transcriptomic, metabolomic and cytokine/chemokine changes in the nose and blood of smokers with Global Initiative for Chronic Obstructive Lung Disease (GOLD, www.goldcopd.org) Stage 2 COPD, healthy smokers with normal lung function and non-smokers, post-cigarette or post-sham/dummy cigarette challenge.

Secondary objectives:

1. To stimulate blood *ex vivo* with IL-1β and PAMPs from smokers with GOLD Stage 2 COPD (16) healthy smokers and non-smokers.
2. To identify molecular biomarkers for COPD patients: to assist in defining novel therapeutic targets, to better stratify phenotypes and to facilitate monitoring of patients.
3. To develop a cigarette smoking challenge model in COPD patients with an aim to utilise this in therapeutic trials of novel therapeutic agents.
4. To carry out platelet aggregometry following stimulation with specific agonists, with the aim of understanding the associated pathophysiology of thrombosis and the pharmacology of respective therapies.

For peer review only

Methods

Study Populations

This is a parallel group study in 3 groups of 12 age, sex, ethnicity, smoking history and BMI matched subjects (Table 1):

- Group 1: Smokers with moderate COPD (GOLD Stage 2)
- Group 2: Healthy smokers with normal lung function (no evidence of COPD)
- Group 3: Healthy subjects who have never smoked

Smokers will be current smokers, smoking at least 5 cigarettes a day, with a minimum pack year history of 10 years. Current cannabis smokers or smokers with a history of moderate or heavy cannabis use will be excluded from the study. Non-smokers must not have smoked a single cigarette in the 12 months prior to the study, and must have smoked less than 100 cigarettes in their lifetime.

Cigarette Smoke Challenge Procedure

One to three screening visits may be required to complete spirometry (and full lung function with gas transfer for carbon monoxide (TLCO) in smokers), 5 slice High Resolution Computerised Tomography (HRCT) (in smokers only) as well as laboratory safety tests, urinalysis, electrocardiogram and physical examination.

On the Cigarette Challenge Day, all subjects will be required to attend our unit at 9am. All subjects must have fasted and refrained from smoking from 9pm on evening prior to the scheduled challenge. Following baseline investigations, smokers in Groups 1 and 2 will smoke 2 cigarettes back to back, in a controlled environment, exhaling the smoke via their nostrils, whilst non-smokers carry out normal tidal breathing over a 10 minute period (Figure 1).

Schedule of Sampling

All subjects will have nasal epithelial curettage and nasosorption procedures with serial blood samples (Figures 2 and 3). Serial nicotine and cotinine levels will also be taken to plot the relative smoke exposure of each individual, as well as providing an objective measure of their baseline smoking habit and clearance of nicotine from their system. Full blood counts and clotting studies will also be performed during the study to ensure patients have not developed any biochemically relevant illnesses or clotting abnormalities.

Analytical Methods

A representative range of non-invasive sampling methods with associated analytical parameters is shown in Table 1.

Nicotine / Cotinine

Nicotine, cotinine and 3-hydroxy-cotinine will be measured in serum over the whole time course. Analysis by capillary gas chromatography will be carried out by Advanced Bioanalytical Service Laboratories (Welwyn Garden City, UK).

For peer review only

8-Isoprostane Enzyme Immuno Assay (EIA)

Measurement of 8-Isoprostane will be carried out in both serum and nasoabsorption fluid at all time points using an 8-isoprostane EIA kit (Cayman Chemicals, Ann Arbor, MI).

Metabolomics

In view of the multiple factors that can influence metabolism, all subjects are required to fast for 12 hours before the challenge begins; during this time they may only consume water. Metabolomic profiling, to be conducted by Metabolon® (North Carolina, USA), measures an extensive range of metabolites (<1000 Da) in plasma and nasal lining fluid after cigarette smoke. Metabolomic analysis will include amino acids, carbohydrates, lipids, nucleic acids and co-factors, molecules of redox homeostasis (e.g. glutathione), organic acids, and small peptides. Importantly, many of the catabolites or biosynthetic intermediates of these metabolites are also detected, assisting in elucidating underlying mechanistic insight. Blood and nasoabsorption samples will be taken at time points listed in Figure 2.

Transcriptomics

Two nasal epithelial curettage samples will be taken using a Rhinprobe™ pre-challenge and at 300 minutes post-challenge. RNA will be extracted using TRIzol® (Invitrogen, Paisley, UK). Blood will be taken at pre-challenge, 20 and 300 minutes post-challenge with RNA extracted using the PAXgene Blood RNA extraction kit (PreAnalytiX GmbH, Hombrechtikon, Switzerland). RNA will be run on an Illumina HT12 v4 (RefSeq Build 38 Rel 22) chip (Illumina, San Diego, USA) with analysis carried out using a dedicated array analysis programme GeneSpring GX 11.3 of all genes. Genes which have changed significantly will be identified using unpaired t-tests ($p<0.05$) with Benjamini-Hochberg False Discovery Rate (FDR) correction. Genes will be reported as 1.2, 1.5 and 2 fold increases. Quality and assessment of global transcriptome changes will be assessed using principle component analysis in GeneSpring. Genes that have changed significantly will also be further explored using a dedicated pathway analysis tool (Ingenuity Systems Pathway Analysis software [IPA]) techniques.

***Ex-vivo* Peripheral Whole Blood Stimulation**

Stimulation of whole blood taken pre-challenge and at 20 and 300 minutes post-challenge will be performed using a range of toll like receptor (TLR) agonists to obtain a 24 hour dose response curve of the following ligands: LPS (TLR4), FSL-1 (TLR6/2), Pam3CSK4 (TLR1/2), Poly(I:C) (TLR3) and IL-1β (Invivogen, San Diego, USA). Serum will then be removed and subsequently measured for CXCL8 and IL-1β by ELISA (R&D Systems, Abingdon, UK).

Platelet aggregation

For platelet aggregation studies, blood will be collected pre-challenge and at 20 and 300 minutes post-challenge. Platelet rich plasma (PRP) will be aliquoted into individual wells of half-area 96-well plates coated with gelatin and one of seven concentrations of arachidonic acid (AA), adenosine diphosphate (ADP), collagen, epinephrine (EPI), ristocetin, TRAP-6 amide or U46619. Platelet aggregation will be determined by changes in light absorbance, and release of thromboxane (TX)A2 by ELISA.

Homogeneous Time Resolved Fluorescence (HTRF) Assay

Serum and nasoabsorption fluid will be screened, across all time points, for Prostaglandin E2 and Leukotriene B4 levels using the HTRF assay kits from Cisbio Assays (Bedford, MA).

Chemokine / Cytokine Immunoassay

Using a Meso Scale Discovery (MSD) immunoassay system (MSD®, Maryland, USA), a variety of chemokines, cytokines and vascular markers will be measured in blood and nasoabsorption samples at the time points list above.

Statistical analyses

This is an exploratory clinical study, and we are aware that there may not be detectable differences between groups based on measurement of particular parameters and the size of effects.

For peer review only

Discussion

Clinical challenge models have been fundamental to clinical research in asthma: employing inhalation of agents such as methacholine, histamine, adenosine monophosphate (AMP), allergens and occupational agents such as isocyanate and ozone. In contrast there has been little clinical research on the effects of cigarette smoke *in vivo* involving patient studies, despite this being the known causative agent in COPD. Development of clinical challenge models that involve cigarette smoke thus have relevance to studying respiratory, cardiovascular and neoplastic effects of cigarette smoke.

The major consideration in developing a cigarette smoke challenge model is the ethical aspect of not encouraging a smoker to continue smoking, and ensuring that maximal support is given to the individual to stop smoking (Table 2). Furthermore, we are studying patients with mild-moderate disease, in whom there may be the possibility of smoking cessation before permanent disability sets in. Other studies have tended to evaluate later stage disease, by which time smoking cessation has less beneficial effects in terms of lung function (53).

A secondary consideration is ensuring the wellbeing of the scientific and clinical staff involved in the study, and minimizing exposure to cigarette smoke. In our study design, all smokers will be established in the habit and will be actively encouraged to enter into a smoking cessation programme immediately following their cigarette challenge. Our unit has adapted a body plethysmography box, with the addition of a carbon filter and HEPA filter, in order to ensure staff are not exposed to the harmful effects of smoke, If subjects were to receive their cigarette challenge outside of the hospital, there may be confounding effects of additional pollutants, temperature and exercise.

An important feature of the study is that smoking two cigarettes is physiologically relevant as a challenge, and that we were able to document levels of nicotine and metabolites over a 5 hour period. We adopted this aspect of the study having considered the design of pharmacokinetic studies with nicotine delivery devices. We intend to standardize the technique of smoking by giving two Marlboro Red cigarettes, noting the total number of inhalations and encouraging exhalation through the nose. Controlled smoke exposure enables accurate assessment of subjects' smoking exposure by measuring concomitant nicotine and cotinine levels. The majority of studies do not mandate any particular smoking restrictions prior to sampling. In this study it may be possible to formally compare nicotine exposure with levels of induced biomarkers.

A key feature of our acute cigarette smoke challenge model involves fasting and refraining from smoking for 12h before and 5h after having a controlled cigarette smoke exposure. We also ensure that patients are not taking any medication that may interfere with responses. This abstinence is necessary due to the extreme sensitivity of measurements such as metabolomics. Metabolomics involves assessment of levels of small molecules and will include molecules such as dietary constituents and drugs. We take serial blood and nasal samples, in a manner similar to a phase I pharmacokinetic study of exposure to a single dose of drug.

For peer review only

The proposed study involves precision nasal sampling. This is non-invasive, has potential for point–of-care, bedside and clinic monitoring. In contrast, bronchoscopy is a research procedure only undertaken with great care in COPD patients. Hence, non-invasive sampling offers great potential for future use of the model in further observational and drug studies. In contrast to many studies looking at gene expression in cross-sectional populations of smokers, our model has the benefit of acquiring samples longitudinally before and after a challenge with a known trigger of the disease. This increases the power to detect effects of the cigarette smoke challenge. We will thoroughly evaluate the acute response to cigarettes with 10 blood and nasal sampling time points within a 5 hour challenge period.

Given the heterogeneity of the disease and the fact that subjects with significant co-morbidities (such as cardiovascular disease) are excluded, we are likely to be evaluating a sub-population of mild COPD patients. This is inevitable in any COPD study, but worth noting, as it may be that this group behaves differently to those with co-morbidities.

The setting up of such studies, using a fully integrated approach that incorporates network biology, where sampling occurs after the administration of causative factors will help us better understand disease and design more robust clinical trials. This type of approach is illustrated in Figure 4 has a far ranging applications to a number of chronic inflammatory diseases.

For peer review only

Contribution statement

Clare L. Ross contributed to designing the clinical aspect of the study and writing the manuscript. Neil Galloway-Phillipps contributed to the ethics submission and design of laboratory-based methods for the protocol. Paul C. Armstrong contributed to the design of the platelet protocols, ethics submission and writing the manuscript. Jane A. Mitchell contributed to the writing of the manuscript and design of the overall study. Timothy D. Warner contributed to the design of the platelet protocols and ethics submission. Christopher Brearley contributed to writing of the ethics, over all protocol design and its compliance with GCP. Mari Ito, Sarah Elkin, Onn Min Kon and Tanushree Tunstall contributed to the design of the clinical protocol, ethics submission and manuscript composition. Trevor T. Hansel was main author contributed to the design of the overall protocol, ethics submission. Mark J. Paul-Clark contributed to the design of the overall protocol, design of the assays, ethics submission and writing the manuscript.

Competing interests

None of the authors that have contributed to this publication have competing interests.

Funding

This study was funded by the Wellcome Trust and Dainippon Sumitomo Pharma Co Ltd, Osaka, Japan.

For peer review only

Table 1.

Summary of Inclusion Criteria and Assessments in the Acute Cigarette Smoke Challenge

**Inclusion Criteria**	**Sample and Assessment Parameters**	**References**
**General criteria for 3 group of subjects, each of 12 individuals**<br>• 45 to 75 years old<br>• Good general health, with no chronic illnesses<br>• No prescribed anti-inflammatory medications (including statins)<br>• Females of childbearing potential have a negative pregnancy test	**Plasma:**<br>Cigarette smoke correlates of exposure<br>• nicotine<br>• cotinine<br>• 3-hydroxycotinine<br>**Plasma** mediators<br>• prostanoids<br>• metabolites<br>• cytokines & chemokines<br>**Whole blood** *ex vivo:*<br>• transcriptomics<br>• TLR-agonist stimulation of leukocytes<br>• platelet aggregation	Nicotine (54)<br><br>Serum cytokines (29;29;55-59)<br><br>Serum metabolites (60-62)<br><br>Blood *ex-vivo* stimulation (16;17)
**Smokers:**<br>Current: ≥5 cigs/day<br>History: ≥10 pack yrs<br>**Non-Smokers:**<br>Have never smoked	**Nasosorption (SAM)**<br>• prostanoids: LTB4, LTC4, PGD2<br>• metabolites<br>• cytokines and chemokines	
**Group 1: Smokers with GOLD Stage 2 COPD**<br>Post-bronchodilator FEV_1 50-79%; Forced Expiratory Ratio <70%<br>TLCO of <80% of normal<br>Emphysema on 5 slice HRCT scan chest	**Nasal curette**<br>• immunohistology<br>• transcriptomics<br>• flow cytometry<br>epithelial culture	
**Group 2: Healthy Smokers**<br>Post-bronchodilator FEV_1 ≥80%; Forced Expiratory Ratio ≥ 70%<br>TLCO ≥ 80% of normal<br>Normal 5 slice HRCT scan of chest	**Exhaled breath condensate (EBC)**	EBC (33;63)
**Group 3: Healthy Non-Smokers**<br>Post-bronchodilator FEV_1 ≥80%; Forced Expiratory Ratio ≥ 70%<br>TLCO and HRCT not done	**Sputum**	Sputum (32;64;65)

Table 2.

Features and Ethical issues with the Acute Cigarette Smoke Challenge

Features of the model	Ethical Issues
**Advantages:**<br>• Human model<br>• Acute-on-chronic inflammation<br>• Serial non-invasive sampling<br>• Combined direct measurement of biomarkers and *ex vivo* stimulation<br>• Limited numbers of strictly defined subjects<br>• Compare with *in vivo* animal models	All subjects must be advised to stop smoking, and offered full clinical, psychological and pharmacological support to carry this out.<br><br>There must be no encouragement for the subject to begin or continue smoking.<br><br>Some frail COPD subjects will have difficulty fasting and refraining from cigarettes for the morning.<br><br>Clinical disease detected through the investigations must be fully treated, regardless of participation in the study.
**Disadvantages:**<br>• Difficulty recruiting a small number of highly defined subjects<br>• Need to validate upper versus lower airway inflammation, including tissue biopsies<br>• Signal parameters must reliably change after acute cigarette smoke exposure<br>• Lung function and CT changes may occur after acute smoke exposure	Some frail COPD subjects will have difficulty fasting and refraining from cigarettes for the morning.<br><br>The subject should not be taking any anti-inflammatory or confounding therapy: therapy must not be withheld.

For peer review only

Figure Footnotes

Figure 1: Cigarette Smoke Challenge Model

HEPA= high efficiency particulate air

Figure 2: Schedule of Assessments

SAM=synthetic absorptive matrix, TLR=Toll-like receptor

Figure 3: Nasal Sampling Methods

Figure 4. Human Integrated Iterative Inflammometry

For peer review only

Reference List

(1) Thun MJ, Carter BD, Feskanich D, Freedman ND, Prentice R, Lopez AD et al. 50-year trends in smoking-related mortality in the United States. N Engl J Med 2013 January 24;368(4):351-64.

(2) Decramer M, Janssens W. Chronic obstructive pulmonary disease and comorbidities. Lancet Respir Med 2013 March;1(1):73-83.

(3) Strulovici-Barel Y, Omberg L, O'Mahony M, Gordon C, Hollmann C, Tilley AE et al. Threshold of biologic responses of the small airway epithelium to low levels of tobacco smoke. Am J Respir Crit Care Med 2010 December 15;182(12):1524-32.

(4) van d, V, Postma DS, Timens W, ten Hacken NH. Acute effects of cigarette smoke on inflammation and oxidative stress: a review. Thorax 2004 August;59(8):713-21.

(5) Lokke A, Lange P, Scharling H, Fabricius P, Vestbo J. Developing COPD: a 25 year follow up study of the general population. Thorax 2006 November;61(11):935-9.

(6) Rennard SI, Vestbo J. COPD: the dangerous underestimate of 15%. Lancet 2006 April 15;367(9518):1216-9.

(7) McDonough JE, Yuan R, Suzuki M, Seyednejad N, Elliott WM, Sanchez PG et al. Small-airway obstruction and emphysema in chronic obstructive pulmonary disease. N Engl J Med 2011 October 27;365(17):1567-75.

(8) Brusselle GG, Joos GF, Bracke KR. New insights into the immunology of chronic obstructive pulmonary disease. Lancet 2011 September 10;378(9795):1015-26.

(9) Churg A, Wright JL. Testing drugs in animal models of cigarette smoke-induced chronic obstructive pulmonary disease. Proc Am Thorac Soc 2009 September 15;6(6):550-2.

(10) Walters MJ, Paul-Clark MJ, McMaster SK, Ito K, Adcock IM, Mitchell JA. Cigarette smoke activates human monocytes by an oxidant-AP-1 signaling pathway: implications for steroid resistance. Mol Pharmacol 2005 November;68(5):1343-53.

(11) Oltmanns U, Chung KF, Walters M, John M, Mitchell JA. Cigarette smoke induces IL-8, but inhibits eotaxin and RANTES release from airway smooth muscle. Respir Res 2005;6:74.

(12) Karimi K, Sarir H, Mortaz E, Smit JJ, Hosseini H, de Kimpe SJ et al. Toll-like receptor-4 mediates cigarette smoke-induced cytokine production by human macrophages. Respir Res 2006;7:66.

For peer review only

(13) Birrell MA, Wong S, Catley MC, Belvisi MG. Impact of tobacco-smoke on key signaling pathways in the innate immune response in lung macrophages. J Cell Physiol 2008 January;214(1):27-37.

(14) Wang JH, Kim H, Jang YJ. Cigarette smoke extract enhances rhinovirus-induced toll-like receptor 3 expression and interleukin-8 secretion in A549 cells. Am J Rhinol Allergy 2009 November;23(6):e5-e9.

(15) Pace E, Ferraro M, Siena L, Melis M, Montalbano AM, Johnson M et al. Cigarette smoke increases Toll-like receptor 4 and modifies lipopolysaccharide-mediated responses in airway epithelial cells. Immunology 2008 July;124(3):401-11.

(16) Paul-Clark MJ, Sorrentino R, Bailey LK, Sriskandan S, Mitchell JA. Gram-positive and Gram-negative bacteria synergize with oxidants to release CXCL8 from innate immune cells. Mol Med 2008 May;14(5-6):238-46.

(17) Wright WR, Parzych K, Crawford D, Mein C, Mitchell JA, Paul-Clark MJ. Inflammatory transcriptome profiling of human monocytes exposed acutely to cigarette smoke. PLoS ONE 2012;7(2):e30120.

(18) Marwick JA, Wallis G, Meja K, Kuster B, Bouwmeester T, Chakravarty P et al. Oxidative stress modulates theophylline effects on steroid responsiveness. Biochem Biophys Res Commun 2008 December 19;377(3):797-802.

(19) Silverman EK, Palmer LJ, Mosley JD, Barth M, Senter JM, Brown A et al. Genomewide linkage analysis of quantitative spirometric phenotypes in severe early-onset chronic obstructive pulmonary disease. Am J Hum Genet 2002 May;70(5):1229-39.

(20) Vulimiri SV, Misra M, Hamm JT, Mitchell M, Berger A. Effects of mainstream cigarette smoke on the global metabolome of human lung epithelial cells. Chem Res Toxicol 2009 March 16;22(3):492-503.

(21) Spira A, Beane J, Pinto-Plata V, Kadar A, Liu G, Shah V et al. Gene expression profiling of human lung tissue from smokers with severe emphysema. Am J Respir Cell Mol Biol 2004 December;31(6):601-10.

(22) Spira A, Beane J, Shah V, Liu G, Schembri F, Yang X et al. Effects of cigarette smoke on the human airway epithelial cell transcriptome. Proc Natl Acad Sci U S A 2004 July 6;101(27):10143-8.

(23) Spira A, Schembri F, Beane J, Shah V, Liu G, Brody JS. Impact of cigarette smoke on the normal airway transcriptome. Chest 2004 May;125(5 Suppl):115S.

(24) Heguy A, O'Connor TP, Luettich K, Worgall S, Cieciuch A, Harvey BG et al. Gene expression profiling of human alveolar macrophages of phenotypically normal smokers and nonsmokers reveals a previously unrecognized subset of genes modulated by cigarette smoking. J Mol Med (Berl) 2006 April;84(4):318-28.

(25) Halappanavar S, Russell M, Stampfli MR, Williams A, Yauk CL. Induction of the interleukin 6/ signal transducer and activator of transcription pathway in the lungs of mice sub-chronically exposed to mainstream tobacco smoke. BMC Med Genomics 2009;2:56.

(26) Schadt EE. Molecular networks as sensors and drivers of common human diseases. Nature 2009 September 10;461(7261):218-23.

(27) Lo Tam Loi AT, Hoonhorst SJ, Franciosi L, Bischoff R, Hoffmann RF, Heijink I et al. Acute and chronic inflammatory responses induced by smoking in individuals susceptible and non-susceptible to development of COPD: from specific disease phenotyping towards novel therapy. Protocol of a cross-sectional study. BMJ Open 2013;3(2).

(28) Woodruff PG. Novel outcomes and end points: biomarkers in chronic obstructive pulmonary disease clinical trials. Proc Am Thorac Soc 2011 August;8(4):350-5.

(29) Verrills NM, Irwin JA, He XY, Wood LG, Powell H, Simpson JL et al. Identification of novel diagnostic biomarkers for asthma and chronic obstructive pulmonary disease. Am J Respir Crit Care Med 2011 June 15;183(12):1633-43.

(30) Han MK, Agusti A, Calverley PM, Celli BR, Criner G, Curtis JL et al. Chronic obstructive pulmonary disease phenotypes: the future of COPD. Am J Respir Crit Care Med 2010 September 1;182(5):598-604.

(31) Vestbo J, Anderson W, Coxson HO, Crim C, Dawber F, Edwards L et al. Evaluation of COPD Longitudinally to Identify Predictive Surrogate End-points (ECLIPSE). Eur Respir J 2008 April;31(4):869-73.

(32) Singh D, Edwards L, Tal-Singer R, Rennard S. Sputum neutrophils as a biomarker in COPD: findings from the ECLIPSE study. Respir Res 2010;11:77.

(33) MacNee W, Rennard SI, Hunt JF, Edwards LD, Miller BE, Locantore NW et al. Evaluation of exhaled breath condensate pH as a biomarker for COPD. Respir Med 2011 July;105(7):1037-45.

(34) Hillas G, Loukides S, Kostikas K, Bakakos P. Biomarkers obtained by non-invasive methods in patients with COPD: where do we stand, what do we expect? Curr Med Chem 2009;16(22):2824-38.

(35) Hurst JR. Upper airway. 3: Sinonasal involvement in chronic obstructive pulmonary disease. Thorax 2010 January;65(1):85-90.

(36) Hakansson K, Konge L, Thomsen SF, Backer V, von BC. Sinonasal inflammation in COPD: a systematic review. Eur Respir J 2013 November;42(5):1402-11.

For peer review only

(37) Hurst JR, Wilkinson TM, Donaldson GC, Wedzicha JA. Upper airway symptoms and quality of life in chronic obstructive pulmonary disease (COPD). Respir Med 2004 August;98(8):767-70.

(38) Hurst JR, Kuchai R, Michael P, Perera WR, Wilkinson TM, Wedzicha JA. Nasal symptoms, airway obstruction and disease severity in chronic obstructive pulmonary disease. Clin Physiol Funct Imaging 2006 July;26(4):251-6.

(39) Caillaud D, Chanez P, Escamilla R, Burgel PR, Court-Fortune, Nesme-Meyer P et al. Association of chronic nasal symptoms with dyspnoea and quality-of-life impairment in chronic obstructive pulmonary disease. Respirology 2014 April;19(3):346-52.

(40) Hurst JR, Wilkinson TM, Perera WR, Donaldson GC, Wedzicha JA. Relationships among bacteria, upper airway, lower airway, and systemic inflammation in COPD. Chest 2005 April;127(4):1219-26.

(41) Hurst JR, Perera WR, Wilkinson TM, Donaldson GC, Wedzicha JA. Systemic and upper and lower airway inflammation at exacerbation of chronic obstructive pulmonary disease. Am J Respir Crit Care Med 2006 January 1;173(1):71-8.

(42) Nicola ML, Carvalho HB, Yoshida CT, Anjos FM, Nakao M, Santos UP et al. Young "healthy" smokers have functional and inflammatory changes in the nasal and the lower airways. Chest 2014 May;145(5):998-1005.

(43) Chanez P, Vignola AM, Vic P, Guddo F, Bonsignore G, Godard P et al. Comparison between nasal and bronchial inflammation in asthmatic and control subjects. Am J Respir Crit Care Med 1999 February;159(2):588-95.

(44) American Thoracic Society Workshop. Immunobiology of asthma and rhinitis. Pathogenic factors and therapeutic options. Am J Respir Crit Care Med 1999 November;160(5 Pt 1):1778-87.

(45) Gelfand EW. Inflammatory mediators in allergic rhinitis. J Allergy Clin Immunol 2004 November;114(5 Suppl):S135-S138.

(46) Chawes BLK, Edwards MJ, Shamji B, Walker C, Nicholson GC, Tan AJ et al. A letter to the editor: A novel method for assessing unchallenged levels of mediators in nasal epithelial lining fluid. J Allergy Clin Immunol 2010;125:1387-89.

(47) Folsgaard NV, Chawes BL, Rasmussen MA, Bischoff AL, Carson CG, Stokholm J et al. Maternal atopic skewing of the neonatal nasal cytokine signature. Am J Resp Crit Care Med 2011.

(48) Nicholson GC, Kariyawasam HH, Tan AJ, Hohlfeld JM, Quinn D, Walker C et al. The effects of an anti-IL-13 mAb on cytokine levels and nasal symptoms following nasal allergen challenge. J Allergy Clin Immunol 2011 October;128(4):800-7.

For peer review only

(49) Nathan RA, Eccles R, Howarth PH, Steinsvag SK, Togias A. Objective monitoring of nasal patency and nasal physiology in rhinitis. J Allergy Clin Immunol 2005 March;115(3 Suppl 1):S442-S459.

(50) Schadt EE, Friend SH, Shaywitz DA. A network view of disease and compound screening. Nat Rev Drug Discov 2009 April;8(4):286-95.

(51) Nicholson JK, Lindon JC. Systems biology: Metabonomics. Nature 2008 October 23;455(7216):1054-6.

(52) Maclay JD, McAllister DA, Johnston S, Raftis J, McGuinnes C, Deans A et al. Increased platelet activation in patients with stable and acute exacerbation of COPD. Thorax 2011 September;66(9):769-74.

(53) Scanlon PD, Connett JE, Waller LA, Altose MD, Bailey WC, Sonia BA et al. Smoking cessation and lung function in mild-to-moderate chronic obstructive pulmonary disease. The lung health study. Am J Respir Crit Care Med 2000 February 1;161(2 Pt 1):381-90.

(54) Benowitz NL, Hukkanen J, Jacob P, III. Nicotine chemistry, metabolism, kinetics and biomarkers. Handb Exp Pharmacol 2009;(192):29-60.

(55) Sin DD, Miller BE, Duvoix A, Man SF, Zhang X, Silverman EK et al. Serum PARC/CCL-18 concentrations and health outcomes in chronic obstructive pulmonary disease. Am J Respir Crit Care Med 2011 May 1;183(9):1187-92.

(56) Cheng DT, Kim DK, Cockayne DA, Belousov A, Bitter H, Cho MH et al. Systemic Soluble Receptor for Advanced Glycation Endproducts Is a Biomarker of Emphysema and Associated with AGER Genetic Variants in Patients with Chronic Obstructive Pulmonary Disease. Am J Respir Crit Care Med 2013 October 15;188(8):948-57.

(57) Faner R, Tal-Singer R, Riley JH, Celli B, Vestbo J, MacNee W et al. Lessons from ECLIPSE: a review of COPD biomarkers. Thorax 2014 July;69(7):666-72.

(58) Celli BR, Locantore N, Yates J, Tal-Singer R, Miller BE, Bakke P et al. Inflammatory biomarkers improve clinical prediction of mortality in chronic obstructive pulmonary disease. Am J Respir Crit Care Med 2012 May 15;185(10):1065-72.

(59) Kim DK, Cho MH, Hersh CP, Lomas DA, Miller BE, Kong X et al. Genome-wide association analysis of blood biomarkers in chronic obstructive pulmonary disease. Am J Respir Crit Care Med 2012 December 15;186(12):1238-47.

(60) Muller DC, Degen C, Scherer G, Jahreis G, Niessner R, Scherer M. Metabolomics using GC-TOF-MS followed by subsequent GC-FID and HILIC-MS/MS analysis revealed significantly altered fatty acid and phospholipid species profiles in plasma of smokers. J Chromatogr B Analyt Technol Biomed Life Sci 2014 September 1;966:117-26.

For peer review only

(61) Ubhi BK, Cheng KK, Dong J, Janowitz T, Jodrell D, Tal-Singer R et al. Targeted metabolomics identifies perturbations in amino acid metabolism that sub-classify patients with COPD. Mol Biosyst 2012 October 30;8(12):3125-33.

(62) Ubhi BK, Riley JH, Shaw PA, Lomas DA, Tal-Singer R, MacNee W et al. Metabolic profiling detects biomarkers of protein degradation in COPD patients. Eur Respir J 2012 August;40(2):345-55.

(63) Fens N, van der Schee MP, Brinkman P, Sterk PJ. Exhaled breath analysis by electronic nose in airways disease. Established issues and key questions. Clin Exp Allergy 2013 July;43(7):705-15.

(64) Qiu W, Cho MH, Riley JH, Anderson WH, Singh D, Bakke P et al. Genetics of sputum gene expression in chronic obstructive pulmonary disease. PLoS ONE 2011;6(9):e24395.

(65) Singh D, Fox SM, Tal-Singer R, Plumb J, Bates S, Broad P et al. Induced sputum genes associated with spirometric and radiological disease severity in COPD ex-smokers. Thorax 2011 June;66(6):489-95.

For peer review only

Fig. 1. Cigarette Smoke Challenge Procedure

All subjects are asked to refrain from smoking for 12h before and 5h after cigarette challenge.
All subjects are fasted and permitted to drink water for 12h before and 5h after cigarette challenge.

Groups 1 & 2: Smokers
On the morning of the challenge, following baseline investigations, each subject is asked to smoke 2 Marlboro Red cigarettes, one after the other, over a 10 min period in a ventilated chamber. They inhale normally, but exhale only through the nose.

Groups 3: Non-Smokers
On the morning of the challenge, following baseline investigations, each subject is asked to breath normally for 10 min in a smoke-free environment.

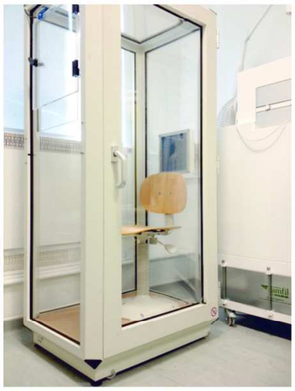

Ventilated chamber with carbon filter attached to ventilation unit in addition to HEPA filter

166x117mm (300 x 300 DPI)

view only

Fig. 2. Schedule of Assessments

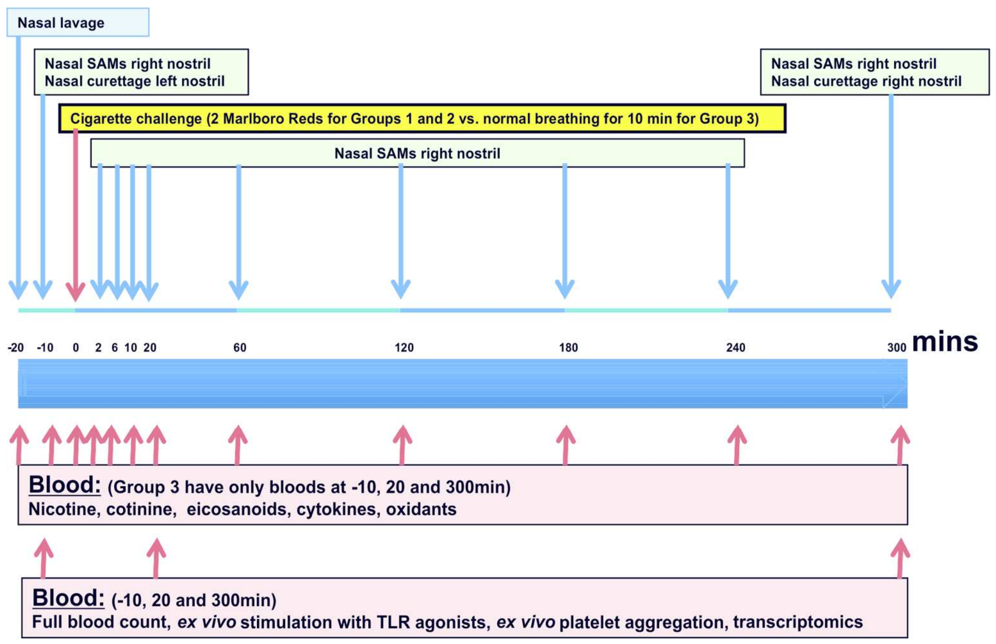

161x119mm (300 x 300 DPI)

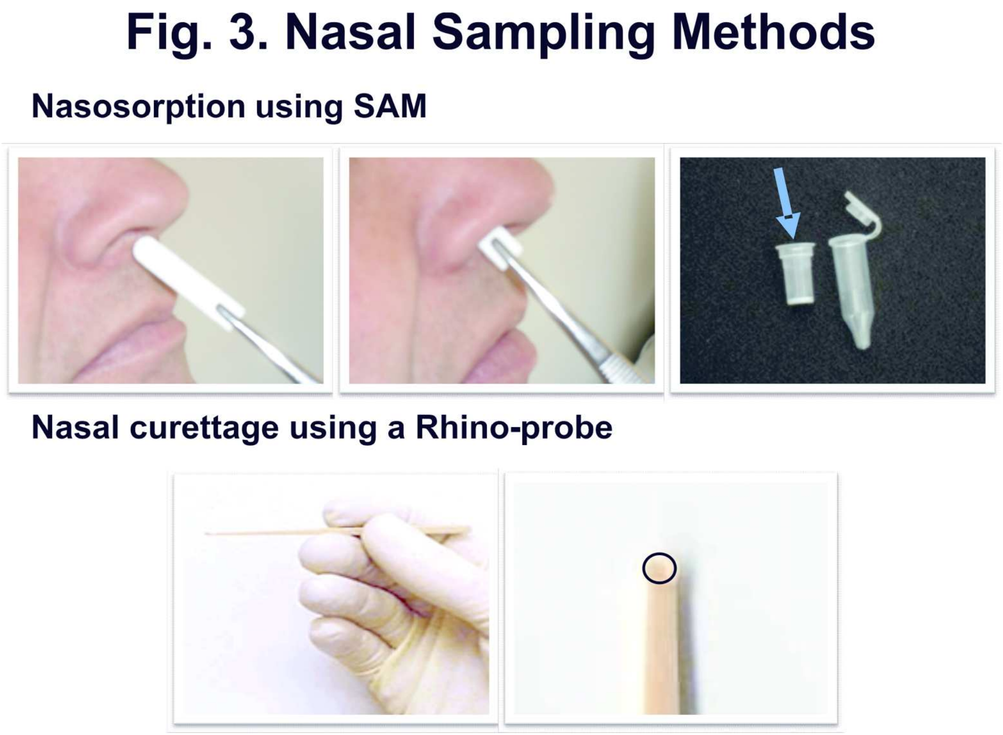

165x119mm (300 x 300 DPI)

view only

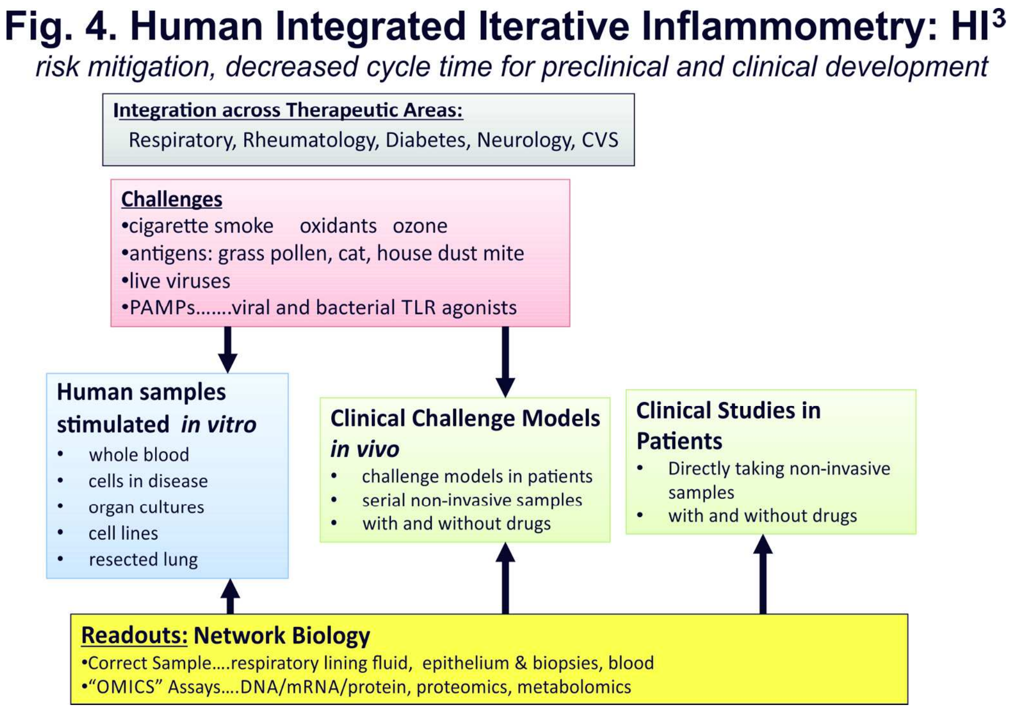

163x113mm (300 x 300 DPI)

view only
